# Hybrid optimization algorithm for enhanced performance and security of counter-flow shell and tube heat exchangers

**DOI:** 10.1371/journal.pone.0298731

**Published:** 2024-03-25

**Authors:** Ajmeera Kiran, Ch Nagaraju, J. Chinna Babu, B Venkatesh, Adarsh Kumar, Surbhi Bhatia Khan, Abdullah Albuali, Shakila Basheer

**Affiliations:** 1 Department of Computer Science and Engineering, MLR Institute of Technology, Hyderabad, Telangana, India; 2 Department of Electronics and Communication Engineering, Annamacharya Institute of Technology and Sciences, Rajampet, Andhra Pradesh, India; 3 Department of Mechanical Engineering, Annamacharya Institute of Technology and Sciences, Rajampet, Andhra Pradesh, India; 4 School of Computer Science, University of Petroleum and Energy Studies, Dehradun, India; 5 Department of Data Science, School of Science, Engineering and Environment, University of Salford, Salford, United Kingdom; 6 Department of Electrical and Computer Engineering, Lebanese American University, Byblos, Lebanon; 7 Department of Computer Networks and Communications, College of Computer Sciences and Information Technology, King Faisal University, Al-Ahsa, Saudi Arabia; 8 Department of Information Systems, College of Computer and Information Science, Princess Nourah Bint Abdulrahman University, Riyadh, Saudi Arabia; Shahrood University of Technology, ISLAMIC REPUBLIC OF IRAN

## Abstract

A shell and tube heat exchanger (STHE) for heat recovery applications was studied to discover the intricacies of its optimization. To optimize performance, a hybrid optimization methodology was developed by combining the Neural Fitting Tool (NFTool), Particle Swarm Optimization (PSO), and Grey Relational Analysis (GRE). STHE heat exchangers were analyzed systematically using the Taguchi method to analyze the critical elements related to a particular response. To clarify the complex relationship between the heat exchanger efficiency and operational parameters, grey relational grades (GRGs) are first computed. A forecast of the grey relation coefficients was then conducted using NFTool to provide more insight into the complex dynamics. An optimized parameter with a grey coefficient was created after applying PSO analysis, resulting in a higher grey coefficient and improved performance of the heat exchanger. A major and far-reaching application of this study was based on heat recovery. A detailed comparison was conducted between the estimated values and the experimental results as a result of the hybrid optimization algorithm. In the current study, the results demonstrate that the proposed counter-flow shell and tube strategy is effective for optimizing performance.

## 1. Introduction

Thermal energy is efficiently transferred using heat exchangers in various engineering applications [1]. Despite their numerous applications, heat exchangers play a vital role in the optimal use of energy resources, from regulating the climate in buildings to converting chemical processes into electricity [2,3]. Owing to their cost-effectiveness, ease of fabrication, and remarkable energy transfer efficiency, double-pipe heat exchangers have gained popularity among the many types of available heat exchangers [4].

Heat exchanger performance optimization is a primary challenge in the field. Researchers have experimented with coiled wires, helical/twisted tapes, wings, and extended surfaces in various ways to improve efficiency [5,6]. Heat exchangers are difficult to optimize when using conventional mathematical models, particularly when dealing with nonlinear relationships and intricate calculations. Alternative methods have been explored as a result of this challenge, with Artificial Neural Networks (ANN) emerging as a promising approach [7–10].

A number of machine learning techniques are available to predict the heat exchanger performance, including ANNs. However, it is challenging to achieve the desired level of accuracy. Innovative methodologies have been proposed for estimating heat transfer rates using artificial neural networks [11,12]. We propose a breakthrough solution to this challenge using hybrid grey neural networks. With this innovative approach, heat exchanger performance can be predicted and optimized more effectively with fewer learning errors [13].

Although engineering advances are incredibly significant, their impact extends far beyond it. Environmental preservation and energy efficiency are globally imperatives. Heat exchangers contribute to substantial reductions in energy consumption and carbon emissions not only by improving the systems into which they are integrated. Sustainable resource management and energy efficiency are of profound importance in an era in which sustainability and responsible resource management are central. Research in this field has had a positive impact on these areas. As a result of our study, there is an urgent need for innovation and improvements in the technology of heat exchangers in this crucial area.

As energy efficiency in heating, ventilation, and air conditioning, chemical processing, and power generation continues to increase, this research takes advantage of this demand. A hybrid method combining grey relational analysis, neural adaptation, and particle swarm optimization was presented to augment counterflow shell-and-tube heat exchangers. NFTool and GRE are advanced tools used in this study to identify critical factors in heat-exchange processes that maximize efficiency and dependability and provide a solution applicable to multiple areas of engineering. As a hybrid methodology, the hybrid methodology synergizes the strengths of each component, resulting in a highly efficient and precise optimization process. In response to global imperatives for energy efficiency and environmental preservation, such research plays a significant role in improving sustainable engineering practices by reducing energy consumption, operational costs, and ecological footprints.

The comprehensive assessment of counter current shell-and-tube heat exchangers (STHEs) are mentioned in Section 2. The methodology, numerical approach and pseudo code of the proposed approach are presented in section 3. Sections 4 provide the experimental design with respect to Heat Exchanger setup specifications. Empirical Investigations of Heat Exchangers is presented in section 5. GRA, NFTool, and PSO based optimization results are presented in Section 6. Section 7 highlights the novelty and potential significance of the proposed integrated approach by considering its significant contributions, future directions and highlights with respect to the novelty and potential significance of countercurrent STHE improvements.

## 2. Related works

In a study by Garcia-Morales et al. [14], inverse artificial neural networks (ICANNi) are proposed to control a heat exchanger. ICANNi control is simple, parameter-independent, and computationally efficient. It outperforms PID and ANNi controllers, achieving faster convergence and no overshoots during reference changes. A mean square error of 0.2025 is

obtained with the ICANNi control after an average establishment time of 23 s. Its flexibility suggests potential application to other systems, warranting further investigation.

A novel metaheuristic approach based on neural networks to predict global gold market signals was introduced by Mousapour Mamoudan et al. [15]. By combining CNN-BiGRU models and allocating influence variables with moth-flame optimization algorithms, they developed a method based on a CNN-BiGRU model optimized using the firefly metaheuristic algorithm. The approach, which can also be used in other precious metals markets, was first created for the gold market to improve forecast accuracy and reduce investor losses.

Ebrahimi-Moghadam et al. [16] performed a thorough hydro-thermal study and optimization for disturbance in nanofluid flow within heat exchangers using helical coil insertion. By analyzing sensitivity and optimizing genetic algorithms (GA), entropy was minimized. It was found that the coil pitch-to-diameter ratio had the most significant impact on thermodynamic properties, followed by the nanoparticle volume fraction. The use of nanoparticles up to 0.02 vol% improved the generation of dimensionless entropy by 13.93%.

The heat exchanger with a corrugated outward surface was optimized using an experimental design using response surfaces by Wei Wang et al. [17]. The focus was investigating complex turbulent flow features and their impact on enhanced heat transfer, particularly on the adjacent shell. The findings have shown that fluid-wall impact significantly improved heat transfer, while spiral flow had minimal effect. An optimum design had a diameter of 38 mm to obtain a high heat coefficient of transfer while maintaining a low-pressure drop. The experiment’s response surface design was used to match the stream rates of the STHE sides. Heat transfer efficiency, energy benefit, and pressure drop were all considered. Four ideal options were presented based on a variety of performance criteria.

According to Azad et al. [18], heat exchangers can be developed using structural theory. This study considered operational and capital costs to reduce the heat exchanger’s total cost. Heat transfer coefficients were improved through construction theory, resulting in reduced capital costs. In addition, frictional pressure loss and pumping energy costs were minimized. Using structural theory, the authors optimized the objective function using a genetic algorithm. According to the case study, 50% of costs can be saved by modifying the design compared to traditional methods. Heat exchangers with shells and tubes are benefited from structural theory.

Tien et al. [19] investigated a spiral-shaped double-pipe heat exchanger. The secondary motion caused by the spiral arrangement improves heat transmission. They evaluated the impact of operational settings on nanofluid heat transfer using Fluent software. In specified Reynolds number ranges, optimal performance was obtained with water-Al2O3 (Aluminium oxide) and water-SiO2 (oxide of silicon) nanofluids. The analysis considered friction coefficient, pressure drop, and thermal performance, with nanoparticle type becoming more significant at higher Reynolds numbers.

Thejaraju et al. [20] thoroughly examine passive improvement approaches in double-pipe heat exchangers. The review includes experimental and numerical studies, analyzing augmented approaches, working conditions, heat transfer enhancement percentages, and working fluids. Various techniques like fins, strip inserts, swirl generators, and coiled wires are examined for their impact on heat transfer performance, highlighting the influence of geometric parameters, material thermal conductivity, and design configurations.

Ebrahimi-Moghadam et al. [21] analyzed methods to improve heat transmission and frictional aspects of double tube heat exchangers (DTHEs). The research concentrated on passive approaches such as turbulator insertion, expanded surfaces, geometry alterations, and nanofluids. The researchers discovered that raising the Reynolds number improved the heat transfer rate. Twisted tape inserts were also helpful. Integrating nanofluids using other methods has promise. Future studies should examine individual approaches, transitory regime effects, and various fluids for work and technique combinations.

Significant cost reductions on a heat exchanger made of shells and tubes (STHX) were realized in a study by Jamil and colleagues [22]. Researchers investigate how various factors affect operational expenditures, including mass flow and baffles. This study provides significant knowledge and views on heat exchangers’ thermal-hydraulic operation and economics.

Abbasi et al. [23] present a novel STHE architecture with sectional plates. They investigate the thermo-hydraulic effects of these baffles using computational fluid computational tools and Supervised Learning approaches. Using multi-objective optimization and empirical research, they discover the ideal design that maximizes heat transmission while minimizing pressure loss. Heat exchanger efficiency can be significantly improved by this innovative design method.

Shahsavar et al. [24] address the urban energy crisis by presenting a novel biogas energy supply framework that is applicable to waste management and green buildings. In their approach, artificial intelligence (AI) techniques such as Random Forest and Artificial Neural Network (ANN) are combined with the Response Surface Methodology (RSM). Accumulated Biogas Production (ABP) can be accurately predicted by ANFIS, which has an impressive correlation coefficient of 0. This study addresses the problems of waste management and bioenergy supply in green buildings, which supports the objectives of sustainable development.

According to Thanikodi et al. [25], a hybrid neural network technique may be utilized to model and anticipate the heat transfer rate in an STHE. Teaching Learning Optimization (TLO) is a strategy for enhancing artificial neural networks (ANN) training. Their findings show that the hybrid technique outperforms traditional methods in prediction accuracy. This study indicates the suitability and flexibility of the suggested approach for heat exchanger development and simulation, contributing to field advancements.

Gholizadeh et al. [26] examined how production management is affected by Electric Discharge Machining (EDM), emphasizing its benefits over conventional techniques. The authors investigated the effects of the electrode corrosion percentage, volumetric flow rate, and surface roughness on the EDM machining parameters. The research forecasts and optimizes EDM parameters and offers insights into manufacturing processes and supply chain applications using a mathematical modeling approach involving an adaptive network-based fuzzy inference system (ANFIS) and Fuzzy Possibility Regression Integrated (FPRI).

Algarni et al. [27] describe a detailed hybrid optimization approach for nano-additives in an STHE system. To improve essential system aspects, they employ experiments design, the computation of fluid dynamics, neural network algorithms and multi-criteria decision-making methodologies. According to the data, thermal conductivity, density, and specific heat they were increased significantly. This shows that advancements in energy storage and phase transition materials are conceivable.

The method presented by Kazi et al. [28] enables the precise design of individual heat exchangers within the network of heat exchangers. Their method entails a multistep procedure that includes sub-optimization processes based on modified MINLP and NLP. This technology assures that the resultant heat exchangers are feasible, reduces nonlinearity, and removes the need for manual intervention. The strategy’s success is proved through examples and comparison with current literature, which contributes to the progress of network fusion and design approaches.

Saffarian et al. [29] compared STHE with varying tube cross-sections and locations. The heat transmission performance was best when elliptical tubes near the shell were combined with circular tubes in the centre. The position of the tubes had a significant influence on heat transmission, with tubes closer to the shell contributing more to overall heat transfer. When elliptical tubes were used instead of circular tubes, pressure decreased on the shell side was higher. The proposed configurations increased heat transport while increasing pressure decreased.

Graphene nanofluids were investigated by Fares et al. [30]. Changes in the nanofluid content, flow velocity, and intake temperature significantly improved the heat-transfer coefficient and thermal efficiency. As a result of this study, nanofluids have the potential to improve the performance of heat exchangers and reduce energy consumption.

Zhan et al. [31] present a hybrid approach for assessing China’s low-carbon transportation infrastructure. Deep learning features are integrated with the CRITIC and DEMATEL methodologies to reduce environmental impact. It provides a quantitative evaluation of low-carbon transportation, highlights important variables, and shows how sustainable transportation policies can be applied both domestically and internationally.

Ghazikhani et al. [32] a post-processing system based on machine learning was presented to improve the forecasting of climate precipitation. Using the random forest algorithm, regression techniques were applied to data from Climate Forecast System Version 2 (CFSV2). In addition to software development, this research’s success in Iran helps with disaster prevention and sustainable development by enabling climate prediction and informed decision-making in weather-dependent industries.

Liang et al. [33] created a cross-corrugated triangle duct heat exchanger model. Configuration parameters were optimized using particle swarm optimization while functions with objectives such as the Colburn, friction, and thermal-hydraulic performance index were considered. The multi-objective PSO optimized entropy production rates and total expenses. Air-to-air heat exchangers were designed and optimized in this study.

Recent advances in heat exchanger design and performance have resulted in the development of various optimization strategies. Analytical methodologies, numerical simulations, and heuristic algorithms such as GA, PSO, and SA are examples of these methods. However, limitations like computationally demanding computations, reliance on correct models, and problems dealing with large and nonlinear systems remain. More study

is required to solve these issues and develop more effective optimization solutions [34]. To circumvent these constraints, hybrid optimization strategies that combine the benefits of many optimization algorithms have been created. Optimization processes that utilize hybrid optimization techniques perform and improve more accurately while requiring less computation and expense [35].

Based on the principles of grey relational analysis, neural adaptation, and PSO, a hybrid optimization approach was applied to counterflow shell-and-tube heat exchangers (STHE). To identify the factors influencing the performance of the exchanger, this study uses a systematic approach. The optimal heat exchanger response can be determined by analyzing the critical factors.

The NFTool was used to estimate the grey relation coefficients of the heat exchanger. These coefficients provide valuable insights into the relationship between the input parameters and grey relational grades. GRE is used to create a multi-factor optimization model that provides a comprehensive understanding of the heat exchanger performance.

Assigning grey relational coefficient values to the effective parameters is a function of the PSO algorithm. The heat exchanger performance was improved by this optimization process. The counterflow shell-and-tube heat exchanger performance can be improved by using swarm intelligence-based algorithms, particularly PSO.

Hybrid algorithms are becoming increasingly popular for solving real-world optimization problems because they can exploit the desirable features of individual algorithms and improve the quality of solutions [36]. The hybrid approach achieves improved performance for counterflow shell-and-tube heat exchangers by combining grey relational analysis, neural adaptation, and particle swarm optimization. PSO and GRE are promising approaches that integrate their individual strengths. NFTool, PSO, and GRE were used together to analyze, model, and optimize heat exchanger data to address the complexity of heat exchanger optimization. A variety of optimization problems can be solved more accurately and efficiently using hybrid methodologies. Because optimization methodologies have matured, a variety of algorithmic techniques have gained importance. Based on the convergence of these methods, future advancements in counterflow shell-and-tube heat exchanger technology are possible.

## 3. Methodology

In this study, a hybrid technique was proposed to enhance the performance of countercurrent STHE. GRE, NFTool, and PSO were used. The grey relational degree is determined using GRE, while the grey relational coefficients are predicted using NFTOOL. The expected coefficients were optimized using PSO. The proposed methodology provides a complete approach for optimizing the heat exchanger performance by integrating various strategies. This enables the discovery of relevant variables, accurate coefficient prediction with NFTool, and design optimization with PSO. There are several applications in which the countercurrent STHE can be improved using this technique.

### 3.1. Numerical approach

Numerical methods can be used to determine a STHE’s efficiency. This analysis defines effectiveness of the STHE and it is premeditated using these formulas [37]:

$$\varepsilon =\frac{Q}{Q_{max}}$$ (1)

To determine the maximum heat transfer rate (Qmax), the logarithmic mean temperature difference (LMTDs) and heat retention rate of the hot fluid were combined. The LMTD can be derived from the subsequent equation, which expresses its relationship [38]:

$${\Delta T}_{lm}=\frac{{\Delta T}_{1}-{\Delta T}_{2}}{\ln\frac{{\Delta T}_{1}}{{\Delta T}_{21}}}$$ (2)

where ΔT1 represents the temperature delta between the hot fluid inlet and the cold fluid outlet, while ΔT2 denotes the temperature difference between the hot fluid outlet and the cold fluid inlet.

The rate of heat capacity (C) is determined by the multiplication of the mass flow rate (m) and the specific heat capacity (Cp) of the hot fluid.

$$C=m\;C_{p}$$ (3)

The actual heat transfer (Q) can be calculated using the overall heat transfer coefficient (U), the effective heat transfer area (A), and the mean temperature difference (ΔTlm) as follows [38]:

$$Q=U\;A\;{\Delta T}_{lm}$$ (4)

The effectiveness (ε) of a program can be described in the following way:

$$\varepsilon =\frac{U\;A\;{\Delta T}_{lm}}{m\;C_{p}\;{\Delta T}_{1}}$$ (5)

In this numerical approach, the values of U, A, m, cp, ΔT1, and ΔT2 can be obtained through experimental measurements or simulation techniques. By evaluating the effectiveness, the efficiency of the counter-flow STHE can be assessed, providing valuable insights for design and optimization purposes.

### 3.2. Pseudocodes for the algorithms

Function hybrid optimization (data_sets, target_data, num_particles, num_dimensions, max_iteration)

# Step 1: Define Objective Function (GRE)

def grey_relational_analysis(parameters):

 # Implement GRE calculations

 # Return grey relational grade

# Step 2: Define Neural Network Model (NFTool)

def neural_network_model (input data):

 # Implement NFTool to predict grey relation coefficients

  # Return predicted values

# Step 3: Define PSO Algorithm

def particle_swarm_optimization(objective function, num_particles, num_iterations):

 # Initialize particle positions and velocities

 # Set personal best positions and global best position

 # Define inertia weight, acceleration coefficients, and maximum velocity

 # Iterate through the specified number of iterations

 for iteration in range(num_iterations):

  # Update particle velocities and positions

   # Evaluate fitness of particles using the objective function

   # Update personal best and global best positions

  # Return the best solution found

# Step 4: Main Optimization Loop

def optimize_system ():

 # Specify problem parameters and bounds

  # Step 4.1: Initialize PSO

best_solution = particle_swarm_optimization(grey_relational_analysis, num_particles, num_iterations)

  # Step 4.2: Use NFTool to predict grey relation coefficients for the best solution

predicted_coefficients = neural_network_model(best_solution)

  # Step 4.3: Evaluate system performance using the optimized parameters

   system_performance = grey_relational_analysis(predicted_coefficients)

    # Return optimized parameters and system performance

# Step 5: Execute Optimization

optimized_parameters,   final_performance = optimize_system()

# Display results

print("Optimized Parameters:", optimized_parameters)

print("Final System Performance:", final_performance)

**Fig 1:**
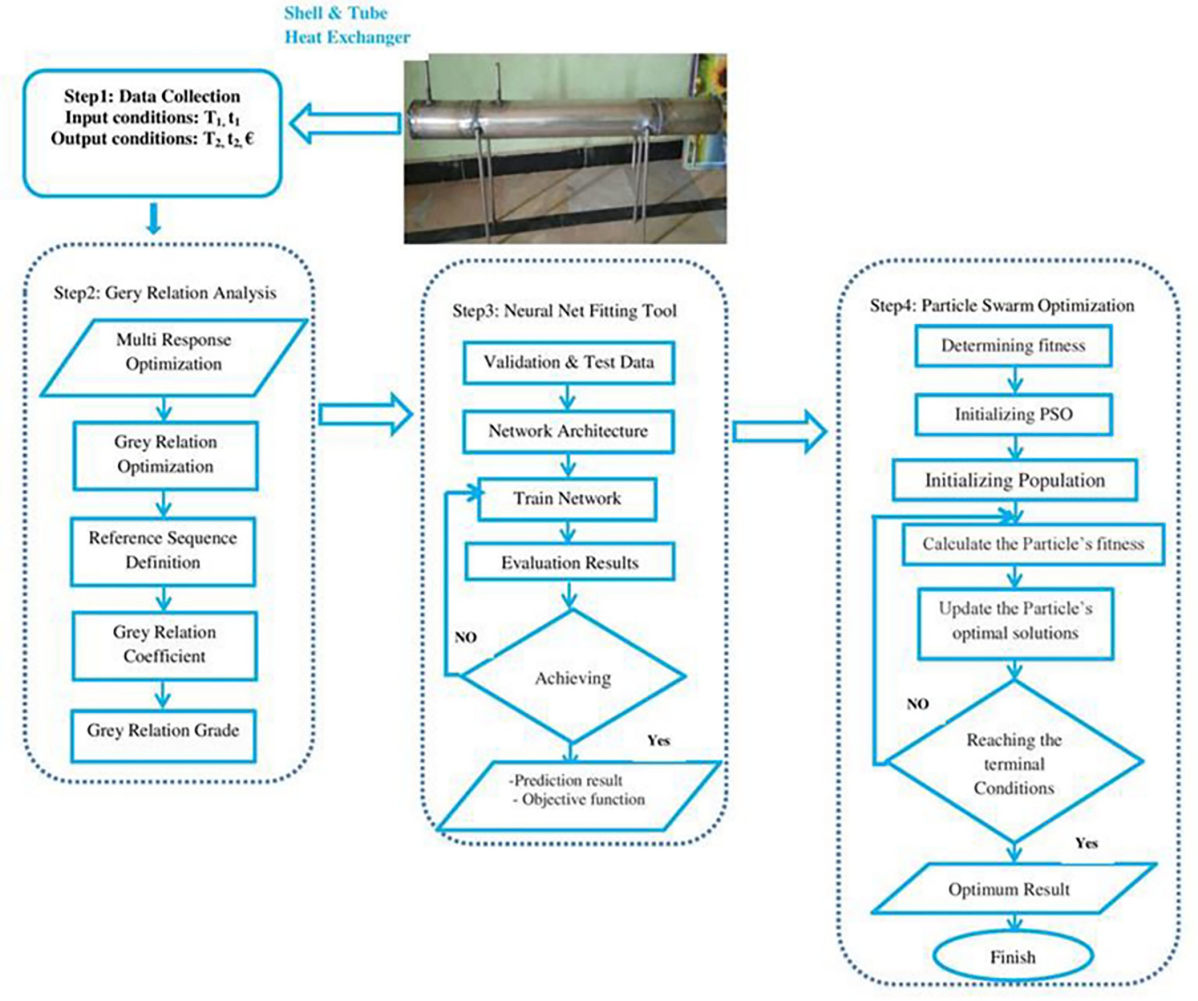
Process flow diagram for hybrid optimization.

A hybrid optimization strategy for countercurrent shell-and-tube heat exchangers was presented in this study. The Process flow diagram for hybrid optimization is shown in Fig 1. This new method provides a condensed representation of the relationships between input parameters by presenting the grey correlation coefficients between them. The neural network training performed by NFTool allows these coefficients to be refined so that they can adapt to intricate patterns. These coefficients were used to conduct a methodological examination of possible configurations indicated by the solution space. The convergence of the iterative process produces the global best particle, which is the best combination of the input parameters. Heat exchanger designs can be fine-tuned to maximize efficiency using this thorough process. By integrating GRA, NFTool, and PSO, the heat exchange systems were optimized significantly. Finally, it contributes to decreased energy consumption, lower operating costs, and minimized environmental impacts. This meets the growing demand for environmentally friendly and energy-efficient heat-exchange systems. This study contributes significantly to the field of heat exchanger optimization and is in line with the sustainability goals of the industry.

## 4. Design of an experiment

**Fig 2:**
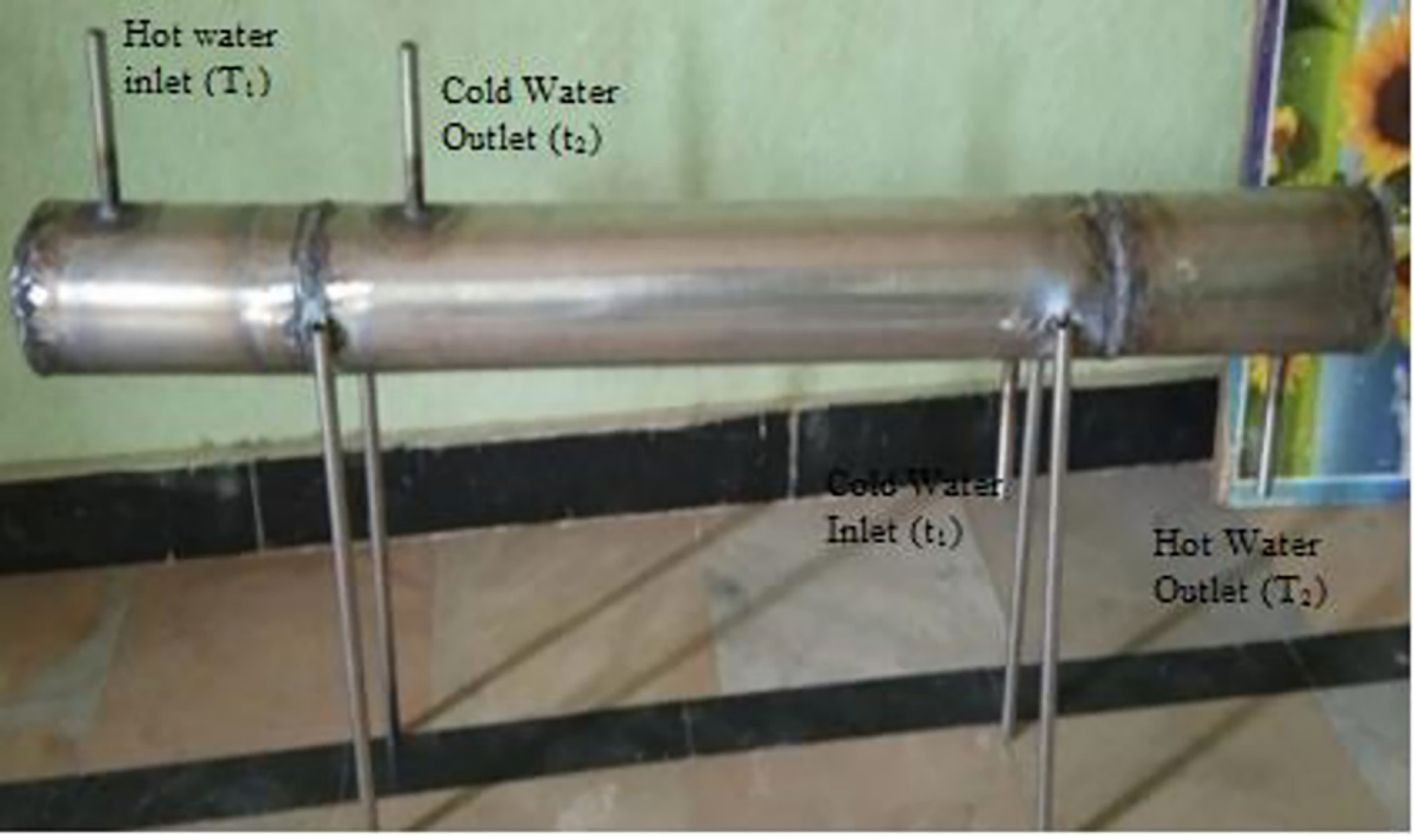
Test configuration of heat exchanger.

This study used a stainless-steel heat exchanger with a single shell, six tubes, and four baffles. The heat exchanger was powered by two motors that circulated the water. This experiment involved the construction of a heat exchanger with the following characteristics. Table 1 lists the details of the heat exchanger. The experimental setup is shown in Fig 2.

**Table 1:** Heat exchanger setup specifications.

Parameters	Value
Physical shape parameter	
Heat transfer characteristics	Indirect contact
Heat exchanger span, L	600 mm
Inner shell size, Di	90 mm
Tube exterior diameter, Do	20 mm
Quantity of tubing, Nt	6
Baffle population, Nb	2
Material class	SS METAL

**Fig 3:**
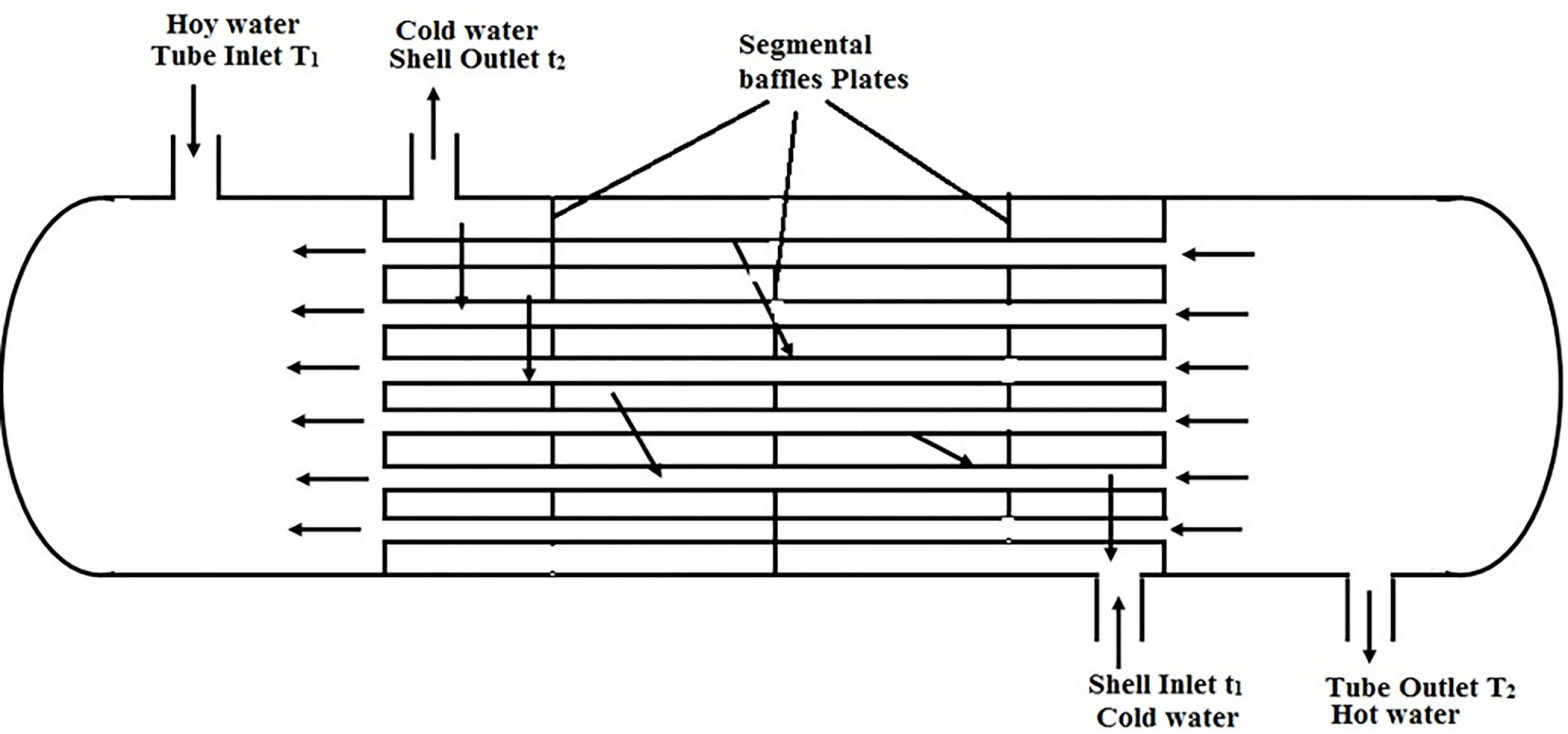
Diagrammatic representation of heat exchanger.

This research cools a high-temperature stream by utilizing both hot and cold water. An STHE circulates cooling water through the shell and hot water through the tubes. Segmental baffles improve heat transport. Laminar counter flow configurations are found to be more efficient than parallel flows. The baffle orientations within the heat exchanger are seen in Fig 3. Baffle spacing is crucial, as higher spacing can lead to less efficient longitudinal flow. Additionally, cross-flow and unsupported tube spans increase the risk of flow-induced vibration and potential tube failure.

Heat transfer coefficients vary by 0.6–0.7 power of velocity on the shell side in turbulent flow (Re > 1,000), whereas pressure drops vary by 1.7–2.0 power of velocity. Laminar flow (Re > 100) has a coefficient of 0.33 and a pressure drop 1.0. When baffle spacing is lowered, pressure drops outpace heat transfer coefficients. Between 0.3 and 0.6 baffle spacing is recommended to ensure efficient heat transfer between the pressure drops and temperatures.

## 5. Empirical investigations of heat exchanger

Multiple variables were considered, encompassing their impacts on output responses, in the Taguchi methodology to optimize the experimental arrangement. The mass flow rates for three distinct test collections ranged from a reduction of 1 kg/min ± 1.42% and 4 kg/min ± 1.42%. Essential factors influencing the output response are summarized in Table 2. Table 3 displays the findings of the experimental investigation on the heat exchanger.

**Table 2:** Test conditions of the heat exchanger experiment.

Attributes	Variables(kg/min)	Grades			
		1	2	3	4
A	Flow rate of hot liquid	1	2	3	4
B	Flow rate of cold liquid	1	2	3	4

**Table 3:** Empirical investigation of the heat exchanger.

S.No.	mh	mc	T1	t1	T2	t2	€
	Kg/min	Kg/min	°C	°C	°C	°C	
1	1	1	80	30	51	59	0.42
2	1	2	75	30	53	52	0.51
3	1	3	70	30	57	47	0.68
4	1	4	65	30	54	44	0.69
5	2	1	75	30	54	49	0.53
6	2	2	80	30	61	48	0.62
7	2	3	65	30	44	47	0.40
8	2	4	70	30	41	53	0.28
9	3	1	70	30	43	63	0.33
10	3	2	65	30	55	52	0.71
11	3	3	80	30	63	55	0.66
12	3	4	75	30	51	48	0.47
13	4	1	65	30	46	58	0.46
14	4	2	70	30	53	55	0.58
15	4	3	75	30	56	47	0.58
16	4	4	80	30	52	58	0.44

Heat is exchanged between the High-Temperature Fluid (HTF) and the Low-Temperature Fluid (LTF) in the exchanger, resulting in a temperature decrease for the HTF (T1 to T2) and an increase for the LTF (t1 to t2). Convection is the primary mode of heat transfer. The HTF and LTF have mass flow rates ranging from 1 to 4 kg/min. Lower HTF flow and higher LTF flow rates show significant temperature variations, indicating improved heat transfer and operational efficiency.

## 6. Optimization results

In this section GRA, NFTool, and PSO are used to optimize the shell and tube heat exchangers. GRA solves single-objective problems using Bayesian regularization, whereas NFTool works with Bayesian regularization. In PSO, important variables are concentrated to maximize gray relationship grades. Using these techniques, the performance of the heat exchangers can be forecasted and maximized. The cost breakdown is briefly discussed along with the economic factors.

### 6.1. GRA-based optimization

GRA integrates qualitative and quantitative data, harmonizing diverse goals and tackling ambiguity in practical scenarios. In engineering, finance, and management science, multi-objective optimization challenges can be solved effectively [39–43].

The goal of this study was to evaluate and optimize variables within a system or process by analyzing gray relations. Several steps were involved in the methodology: (1) normalizing and preprocessing raw data to create grey relations, (2) determining deviation sequences using Eq (8), (3) comparing the normalized result with an ideal reference with Eq (9) to calculate the Grey Relational Coefficient (GRC), and (4) computing the Grey Relational Grade (GRG) by averaging the GRCs obtained from multiple runs using Eqs (6) and (7). Using this approach, variable significance can be assessed, and optimization can be more efficient.

$$Y_{1}(k)=\frac{Max(Y(k))-(Y(k))}{\left(Max\;Y(k))-(Min\;Y(k)\right)}$$ (6)

$$Y_{1}(k)=\frac{\left(Y(k))-(Max\;Y(k)\right)}{\left(Max\;Y(k))-(Min\;Y(k)\right)}$$ (7)

$$\Delta_{0,i}(k)=|{(Y}_{0}^{*}(k))–(Y_{l}^{*}(k))$$ (8)

$$\xi_{0,i}(k)=\frac{\Delta \min+\zeta .\Delta max}{\Delta_{0,I}+\zeta .\Delta max}$$ (9)

$$\gamma i=\frac{1}{p}\sum_{k=1}^{p}\xi_{0,i}(k)$$ (10)

This study employed Grey Relational Analysis (GRA) for single-objective optimization, normalizing the analytical response (Eq 6) between 0 and 1. The method aimed to maximize T2, t2, and €. GRC (Grey Relational Coefficient) (Eq 9) compared absolute values to idealized values using an identification coefficient (ranging from 0 to 1). A commonly used value is 0.5, with minimal impact on parameter significance order in GRA.

In Eq 10, varying weight factors were utilized to compute the Gray Relational Grade (i) by evaluating the correlation between the reference and comparison sequences based on the Grey Relational Coefficient (GRC). A GRC value of 1 indicates identical sequences, and the grey comparison grades are determined by selecting the maximum value from T2, t2, and €. Weighting responses is crucial in GRA as relevance can differ in real-world engineering scenarios. Calculating the weighting factors using an appropriate approach is important to ensure reliable results when considering T2, t2, and other influential parameters.

Genetic response levels (GRGs) were calculated by averaging the GRCs for each response shown in Table 4.

**Table 4:** GRC and GRG for all response variables.

S. No.	Grey relation coefficient			GRG
	GRC-T2	GRC-t2	GRC-€	
1	0.52	0.39	0.61	0.51
2	0.48	0.54	0.48	0.50
3	0.41	0.76	0.35	0.51
4	0.46	1.00	0.34	0.60
5	0.46	0.66	0.46	0.53
6	0.35	0.70	0.39	0.48
7	0.79	0.76	0.64	0.73
8	1.00	0.51	1.00	0.84
9	0.85	0.33	0.81	0.66
10	0.44	0.54	0.33	0.44
11	0.33	0.46	0.36	0.39
12	0.52	0.70	0.53	0.59
13	0.69	0.40	0.54	0.55
14	0.48	0.46	0.42	0.45
15	0.42	0.76	0.42	0.53
16	0.50	0.40	0.57	0.49

### 6.2. NFTool optimization

The NFTool in MATLAB is a simple interface that facilitates the design, training, and analysis of neural networks for various applications, including regression, classification, and time-series prediction. It provides a range of network architectures that allow users to specify the number of layers, neurons, and activation functions. The tool provides visualizations including training curves and error histograms to evaluate the performance of trained networks. In addition, NFTOOL provides data import and pre-processing capabilities that allow users to process various data formats and perform necessary transformations. In addition, cross-validation procedures are recommended to evaluate the generalization

performance of trained models. Using GRC in heat exchanger applications, NFTool can estimate GRG using GRA.

**Fig 4:**
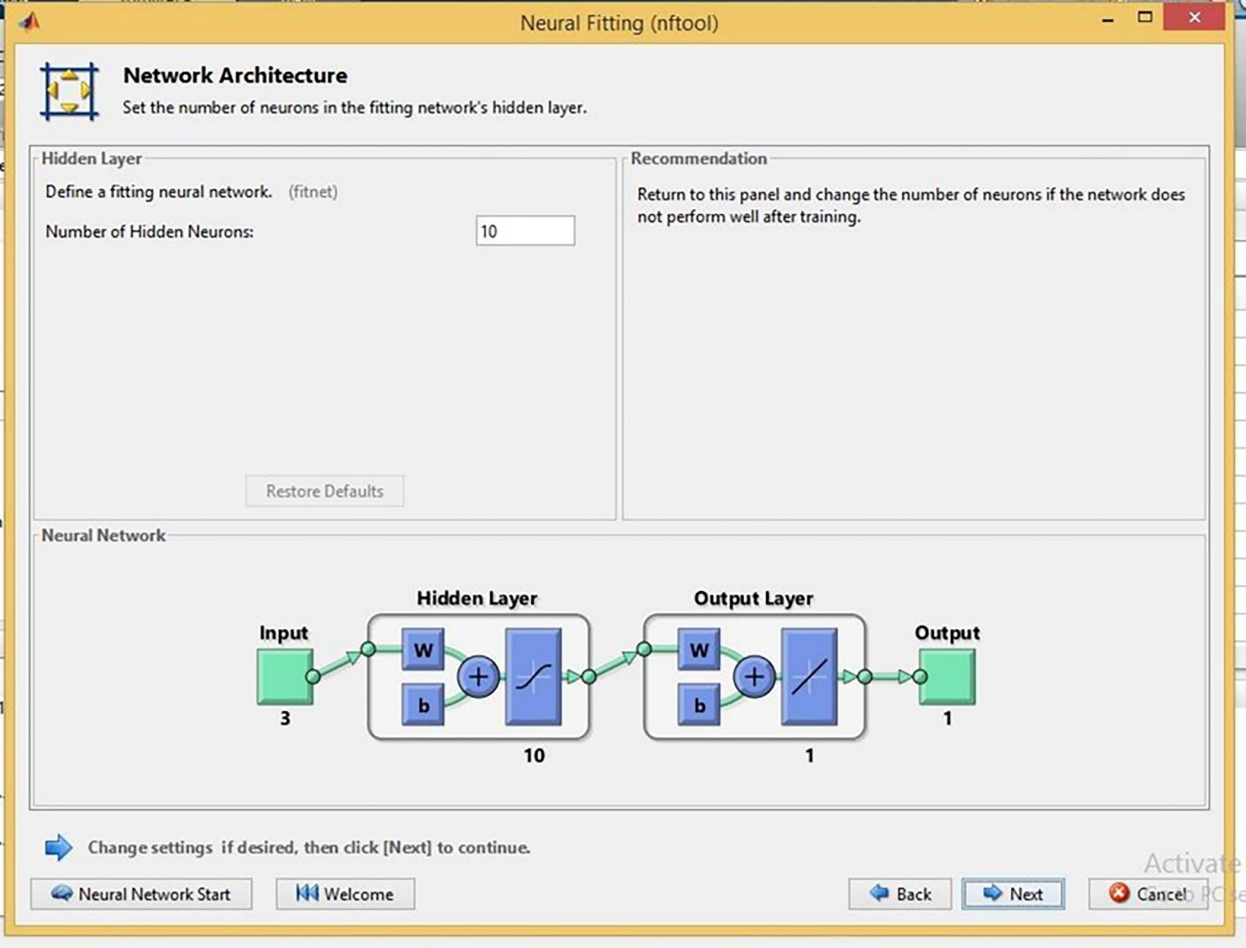
Neural fitting architecture.

**Fig 5:**
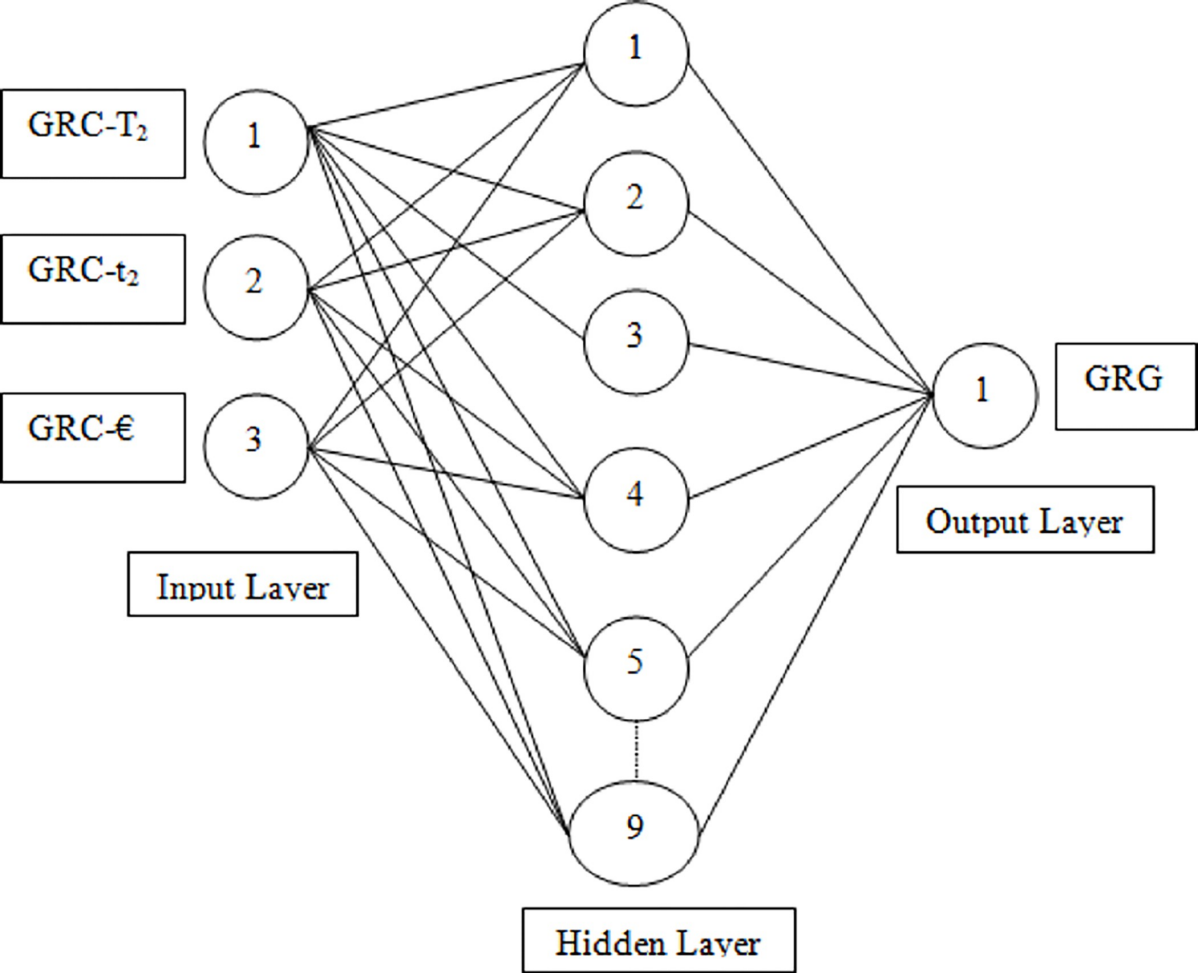
NFTool architecture.

**Fig 6:**
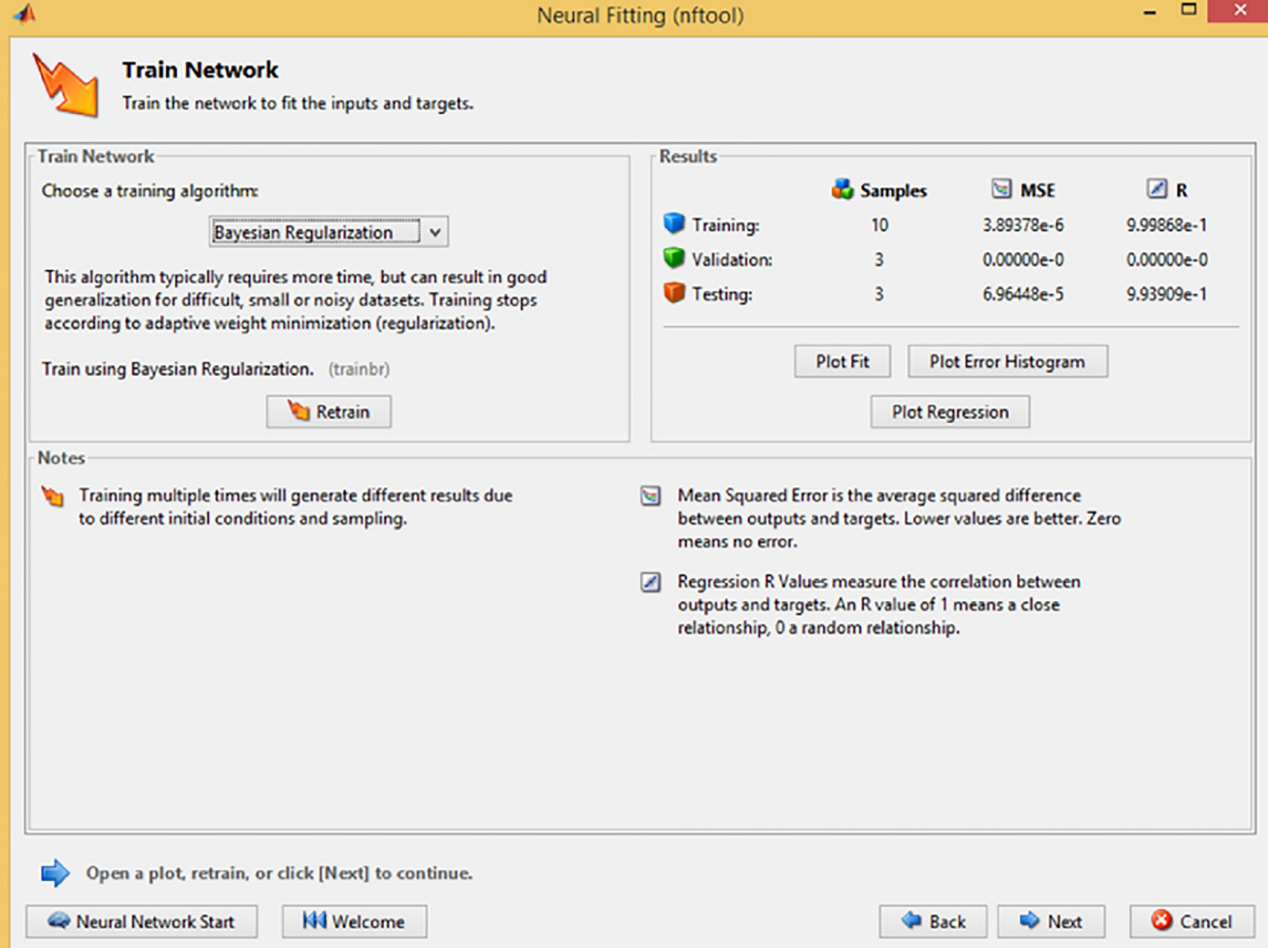
Train network.

**Fig 7:**
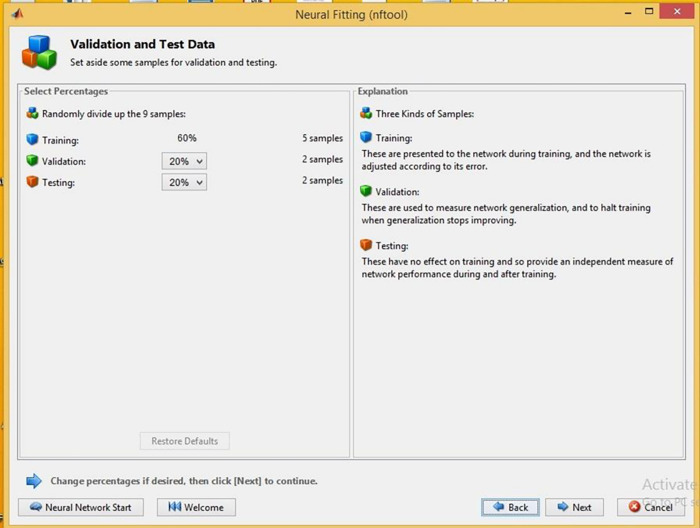
Validation and test data.

During the NFTOOL validation process, the 16 samples were divided into three sets: training, validation, and testing. Training samples accounted for 60%, validation samples for 20%, and testing samples for 20%. This Figure illustrates how the fitting neural network defined the hidden neurons. As illustrated in Fig 4, Bayesian regularization was used as the training algorithm. NFTool’s architecture is shown in Fig 5. Using Bayesian regularization helps improve the model’s generalization capability and prevents over-fitting. The resulting neural network model was then validated and tested using the partitioned data shown in Fig 6. These steps, depicted in Figs 4, 6 and 7, demonstrate the process of using NFTOOL to develop and validate an accurate neural network model for predicting the Gray Relational Grade (GRG) in the context of heat exchanger applications.

**Fig 8:**
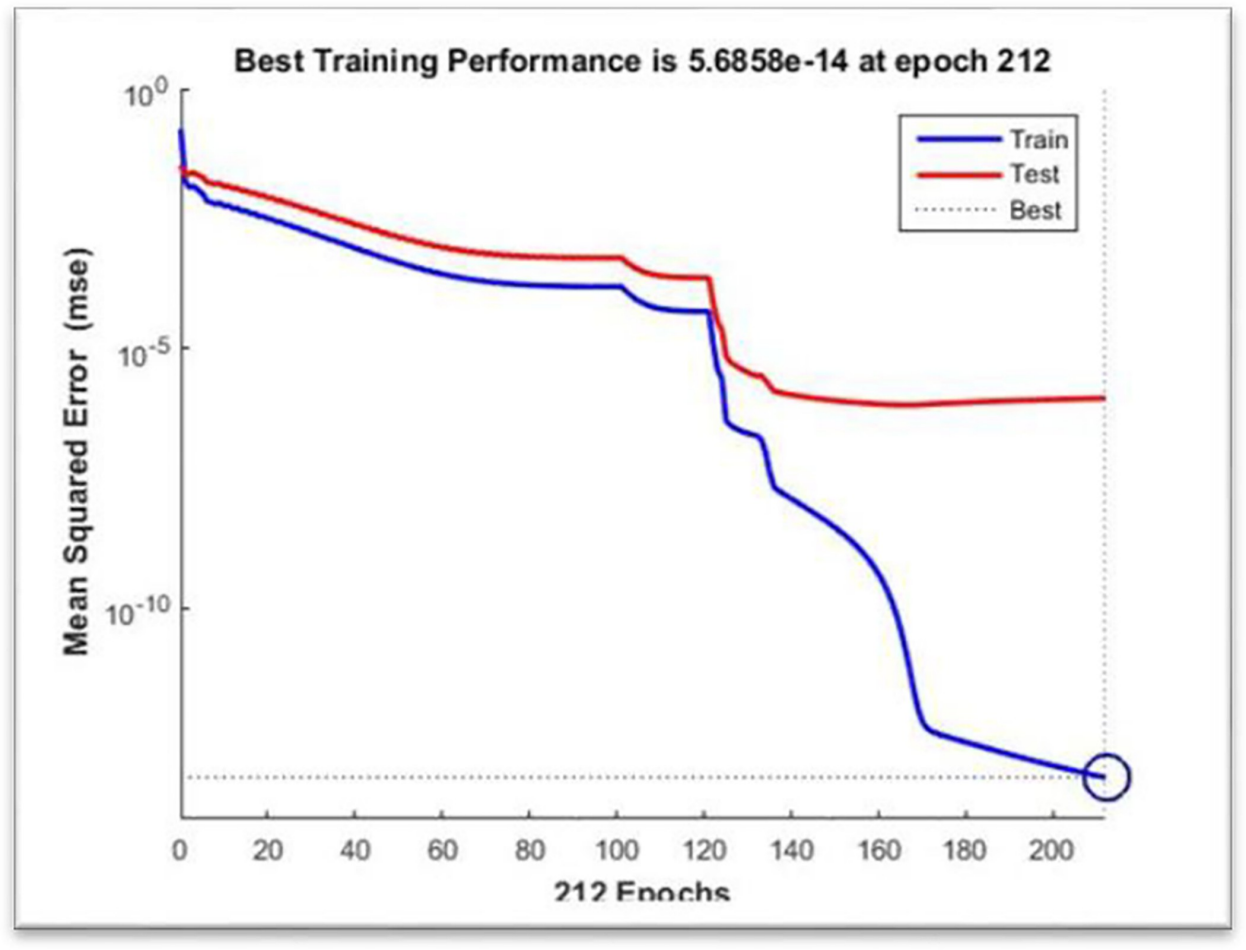
Performance during training.

Fig 8 depicts the training box used in the Neural Fitting Tool (NFTool), which gives vital information into a neural network model’s correctness and performance. After training, the model’s efficacy is assessed using performance metrics, regression results, and error histograms supplied by the tool.

The performance metrics, such as mean squared error (MSE), root mean squared error (RMSE), mean absolute error (MAE), or the coefficient of determination (R^2), quantify the quality of the model’s predictions and its overall performance. These metrics are calculated using the predicted outputs (ŷ) and the corresponding target outputs (y). For example, MSE is computed as:

MSE=(1/n)*Σ(ŷ‐y)^2 (11)

where n represents the data point count. Smaller MSE values indicate stronger correspondence between the forecasted and desired outcomes.

Regression coefficients offer valuable insights into the correlation between the anticipated outputs and the desired outputs. The regression equation expresses this association and can be formulated as:

ŷ=b0+b1*x1+b2*x2+…+bn*xn (12)

where ŷ denotes the forecasted output, b0 represents the intercept, and b1, b2,…, bn symbolize the regression coefficients, while x1, x2,…, xn signify the input variables.

Using histograms to visualize error distributions between predicted and target outputs, it visualizes errors distribution. Calculate the error (e) by making the following comparison between the predicted output (ŷ) and the target output (y):

e=ŷ−y (13)

When examining the error distribution, it becomes easier to identify biases or patterns in model predictions.

Researchers can evaluate the accuracy and reliability of their trained neural network models using performance metrics, regression values, and error histograms as shown in the figures. By providing comprehensive insight into the performance of the model, further improvements or adjustments can be made to improve its predictive capabilities.

Training and validation errors are plotted visually in NFTOOL as a function of training epochs. This helps evaluate the convergence and generalization capabilities of the neural network model. By monitoring error trends, researchers can make informed decisions about model adjustments and optimization, which helps avoid overfitting and achieve better normalization of unseen data. Overall, as shown in Fig 8, performance plots are valuable tools for evaluating and improving the performance of neural network models in NFTOOL.

**Fig 9:**
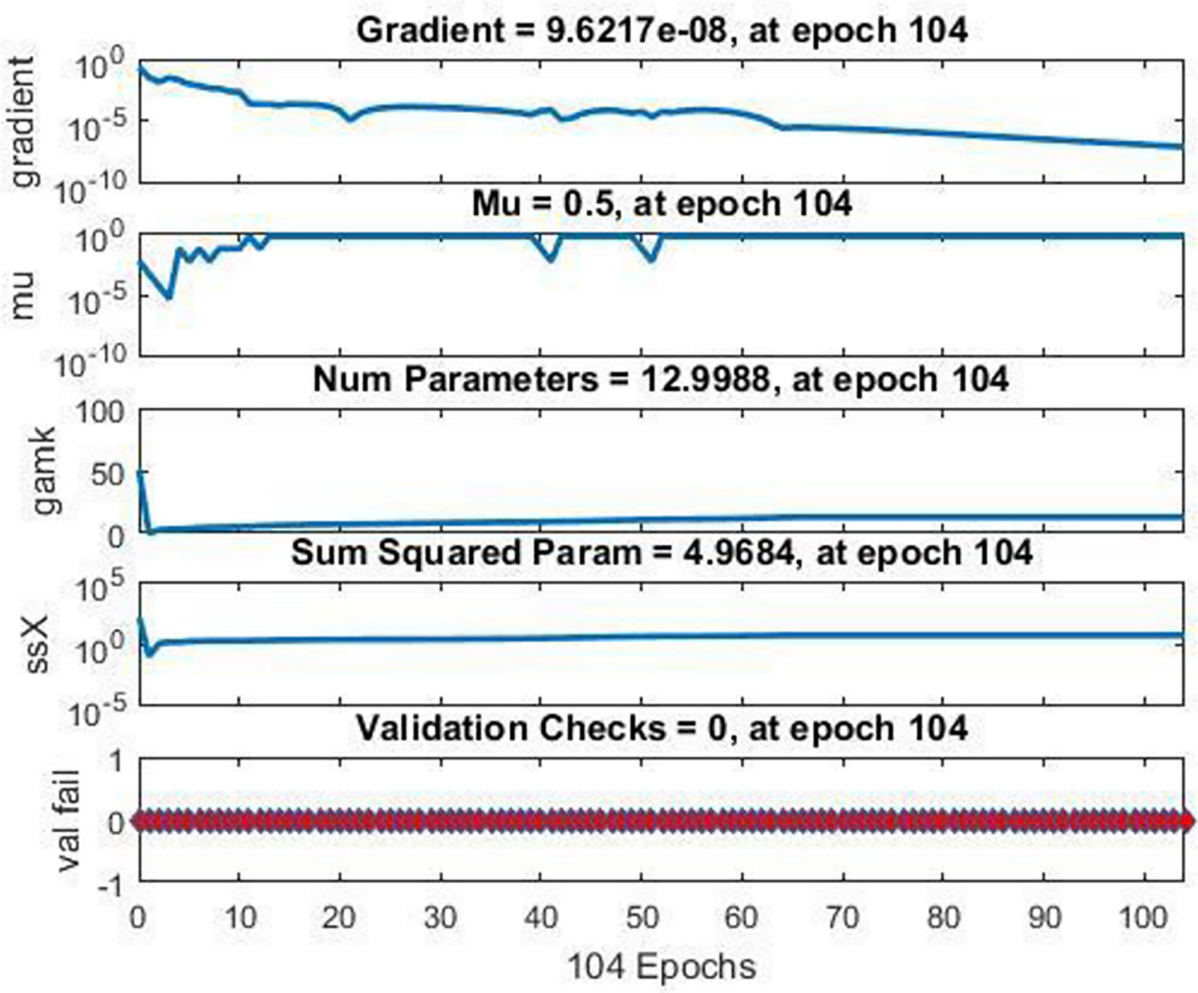
NFTool’s training state.

In Fig 9, NFTOOL highlights the importance of monitoring network progress and performance during training. This graph provides important information including the number of epochs, training errors, and validation errors. The training state analysis facilitates the optimization of the training algorithm and increases the overall training progress by evaluating the convergence and performance of the network. Because of this detailed examination of the training state, neural network models can be developed for a wide range of applications that are more accurate and effective.

**Fig 10:**
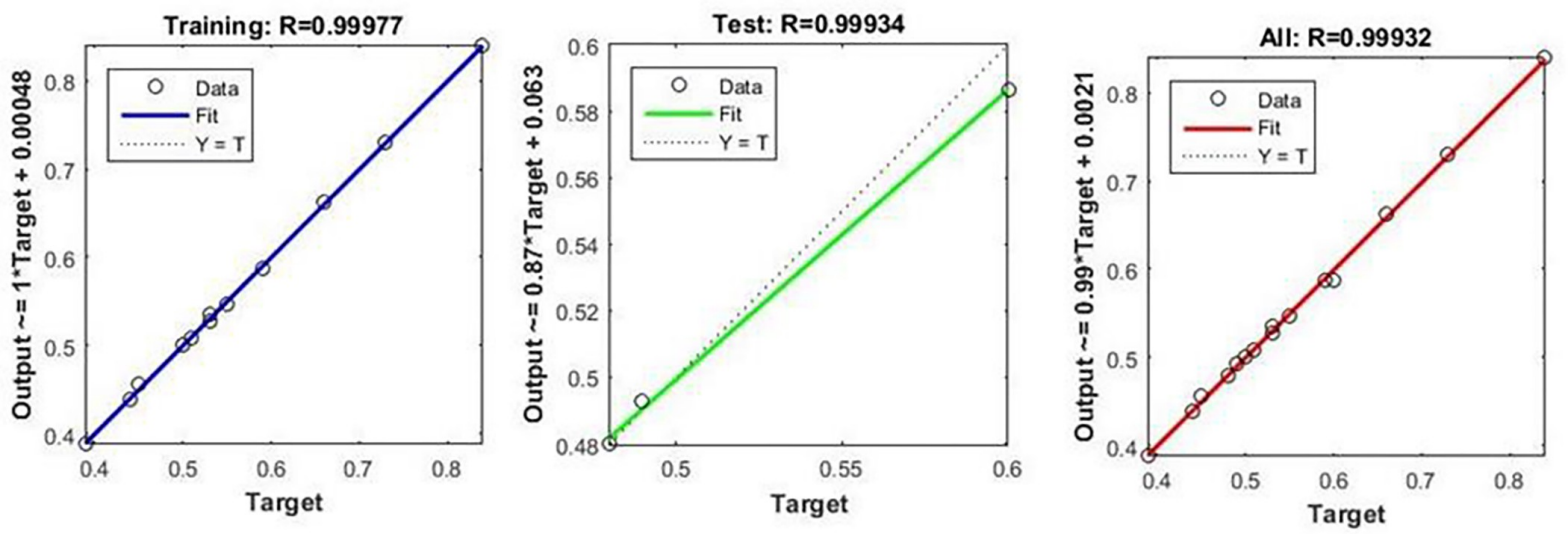
Regression.

Regression analysis should be conducted in NFTOOL to determine the efficiency of the trained neural network. This is the case, as shown in Fig 10. By comparing the predicted outputs with the actual outputs, researchers can assess the accuracy of the model. The predicted and target values were plotted in a regression plot. This allowed us to gain insight into the accuracy and consistency of neural networks. An analysis of this type helps determine whether the trained model is effective and identify potential discrepancies. Model refinement and optimization can be effectively improved by analyzing the regression plot, resulting in improved model reliability and performance.

**Fig 11:**
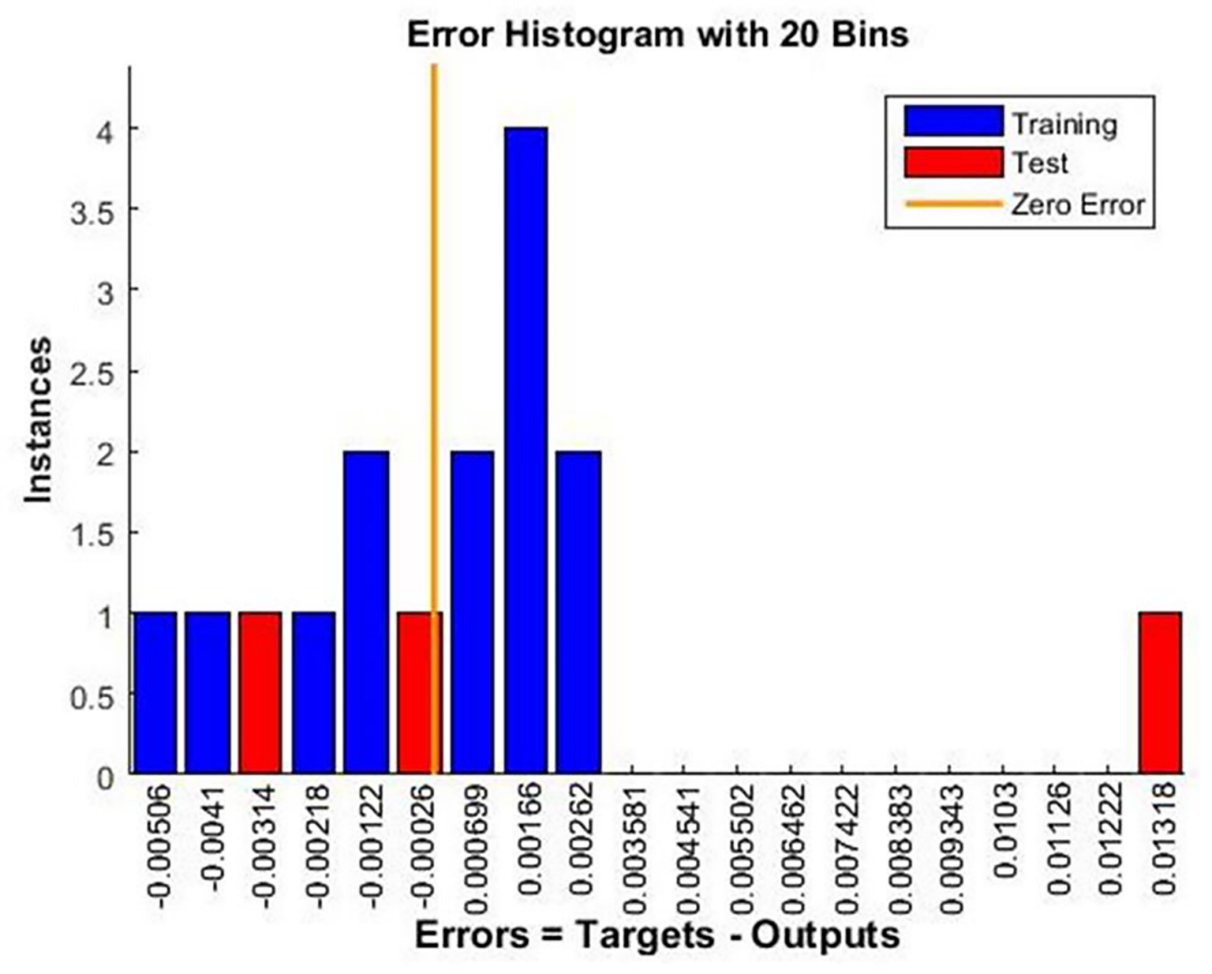
Error histogram.

A histogram of the error distribution between the predicted and actual values is shown in Fig 11. Error characteristics such as bias or skewness can be used to identify patterns or outliers. This analysis helps fine-tune the model for better predictive capability. An error histogram is a valuable tool for evaluating the reliability and effectiveness of a trained neural network model for capturing underlying data relationships.

**Fig 12:**
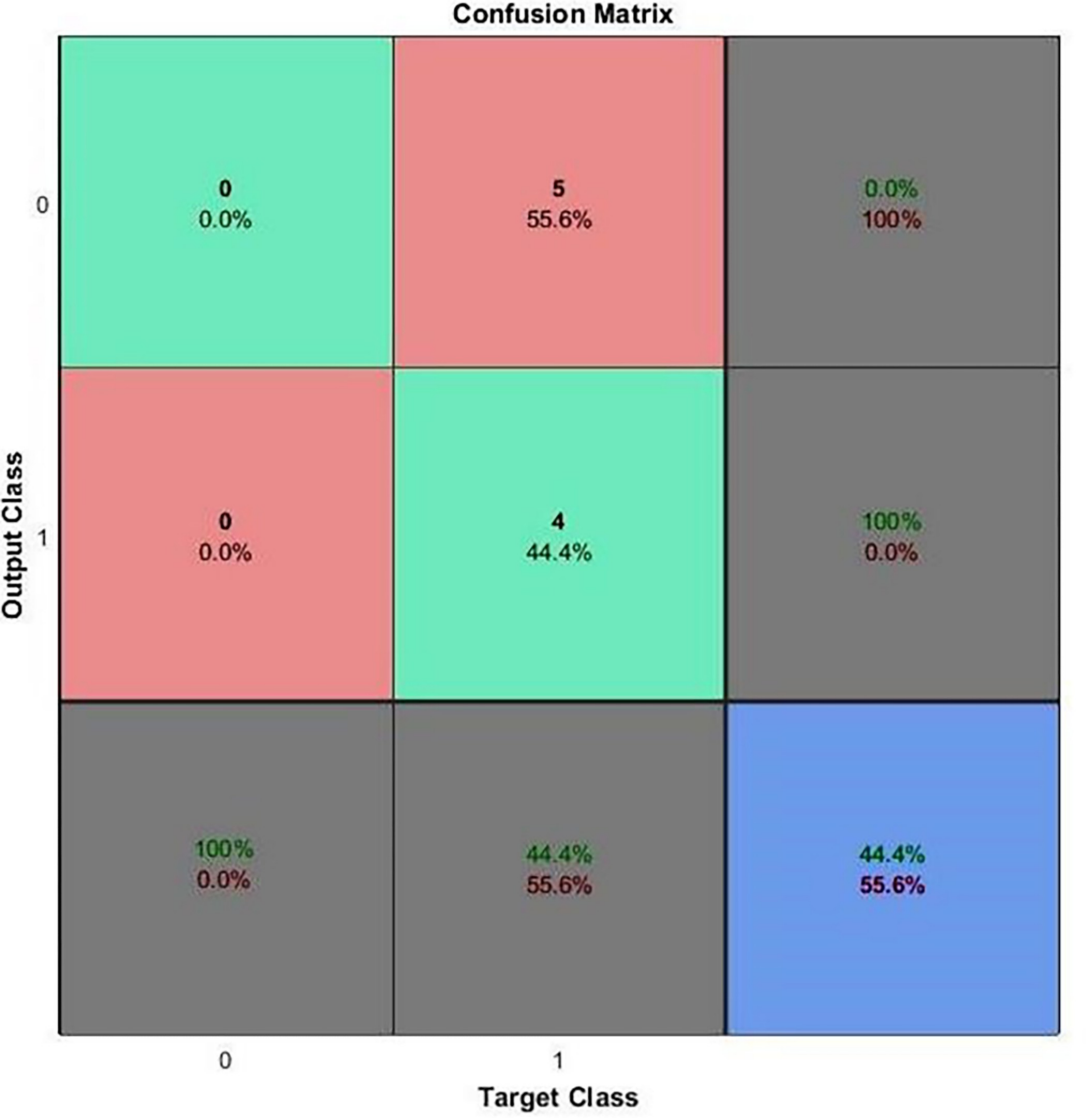
Confusion matrix.

NFTOOL generates classification models such as neural networks using confusion matrices, as shown in Fig 12. This allowed the accuracy of the classification model to be tested by generating a tabular summary of the prediction and actual class labels. The confusion matrix was divided into four categories: (2) true positives, (2) true negatives, (3) false positives, and (4) false negatives. Using this information, the model can accurately categorize instances. Precision, recall, and F1 score are metrics used to measure classification performance; to improve the accuracy of the model’s classification, patterns and biases can be identified in the confusion matrix.

**Fig 13:**
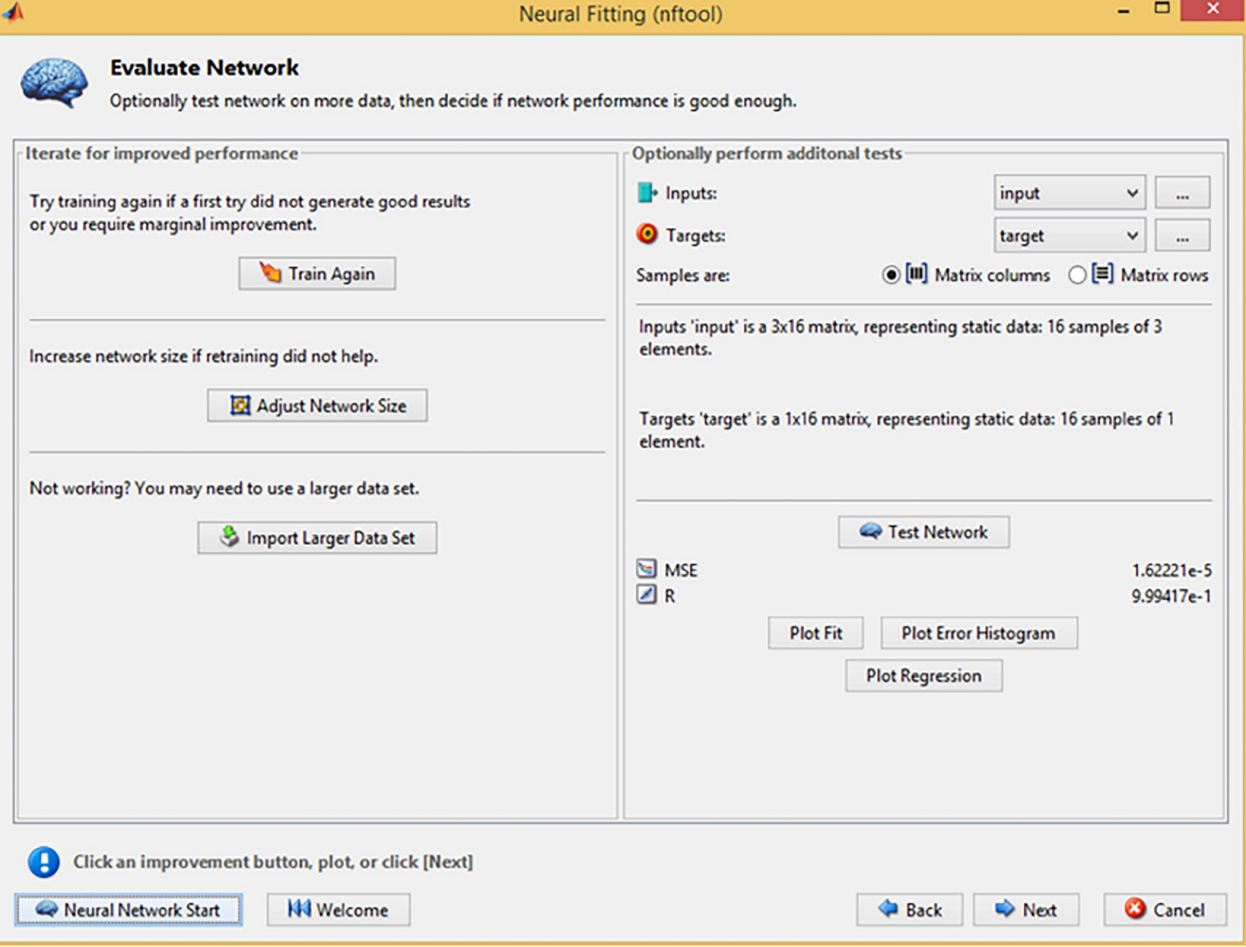
Evaluation results of the trained neural fitting.

Fig 13 illustrates the ease with which the trained networks can be evaluated using NFTOOL. Various evaluation metrics can be obtained after training, using an evaluation function. Using this function, we can calculate the MSE, MAE, and RMSE of a network, which can be used to assess its performance. These evaluation metrics can be used to measure the accuracy and prediction errors of trained networks.

**Fig 14:**
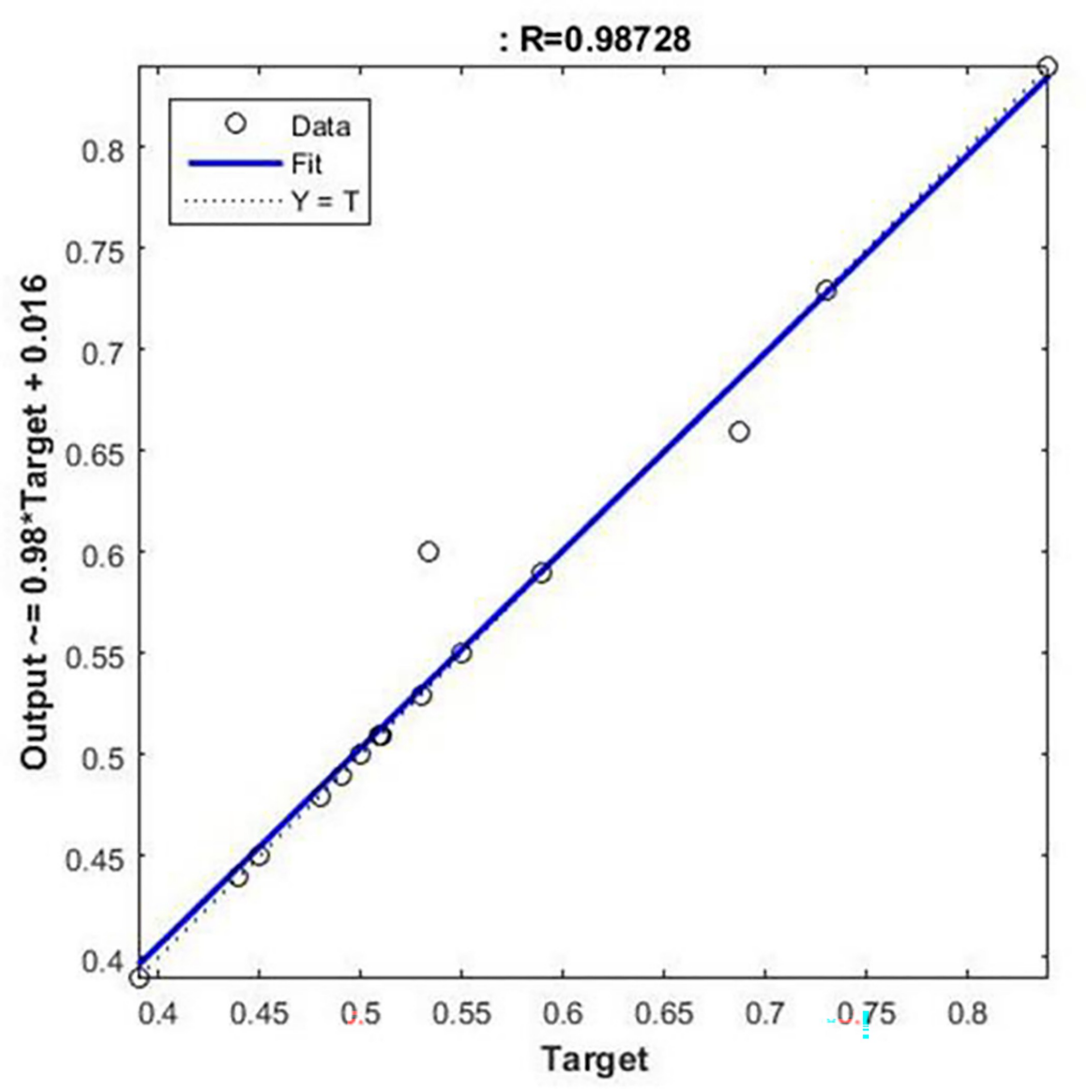
Regression graph for evaluating the trained network.

Based on the training neural network model evaluated using NFTOOL, Fig 14 shows a regression graph of the predicted versus actual values. By comparing how well the model fits the data to the target, this graph shows how accurate the variable prediction is by comparing how well the model fits the data.

**Fig 15:**
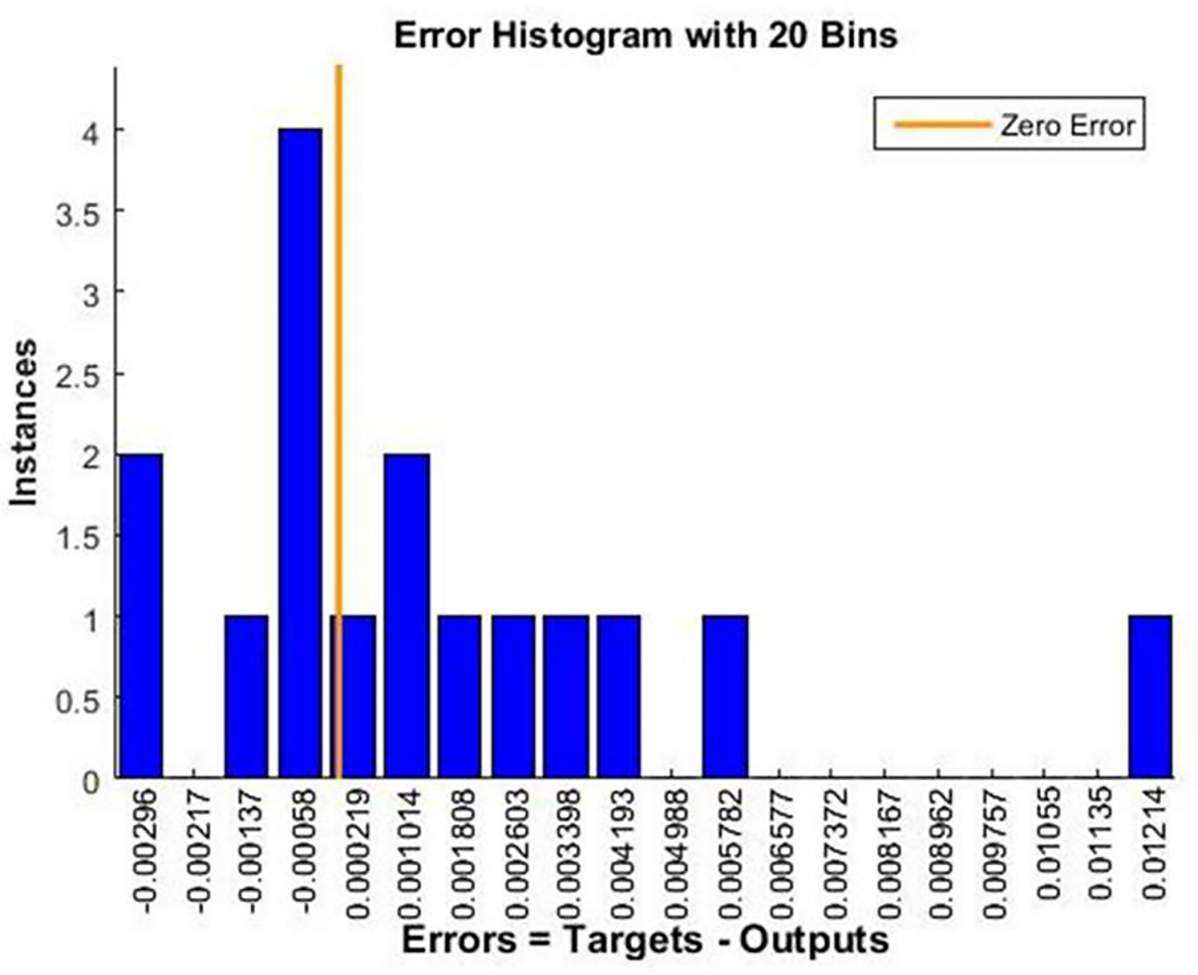
Error histogram for evaluating the trained network.

In Fig 15, the error histogram shows how the trained network made predictions with different errors. An analysis of this histogram provides insight into the magnitude and frequency of errors, providing a picture of how well the network performs in terms of predicting and allowing for the possibility of bias or skew.

In Table 5, the Training and testing were used to develop the NFTool model. For each response, NfTool predicted significant values that agreed with the measurements.

**Table 5:** GRG VS predicted GRG.

	Target	Predicted	
Exp. No.	GRG	GRG	Error
1	0.51	0.51	5.6E-08
2	0.50	0.50	5.3E-08
3	0.51	0.51	1.3E-03
4	0.60	0.53	6.6E-02
5	0.53	0.53	3.9E-07
6	0.48	0.48	1.5E-07
7	0.73	0.73	1.2E-07
8	0.84	0.84	-8.3E-08
9	0.66	0.69	-2.8E-02
10	0.44	0.44	2.2E-07
11	0.39	0.39	2.0E-07
12	0.59	0.59	1.0E-07
13	0.55	0.55	9.1E-08
14	0.45	0.45	5.6E-08
15	0.53	0.53	1.7E-07
16	0.49	0.49	8.4E-08

### 6.3. Particle Swarm Optimization (PSO) algorithm

The grey relational grades of the heat exchanger can be optimized using PSO in this section. It optimizes the grey relational coefficients of the hot and cold outlet temperatures (T2) as well as effectiveness (€) in order to maximize the grey relationship grade. Here are the implementation steps for PSO [44,45]:

#### 6.3.1. Initialization

A particle population is initialized by the PSO algorithm. Depending on T2, t2, and €, each particle represents a potential solution.

#### 6.3.2. Evaluation

Heat exchanger GRG are used to evaluate particle fitness. The GRG measures the similarity between the particle’s performance and the best performance observed so far.

#### 6.3.3. Update particle position and velocity

PSO equations are used to iteratively update particle positions and velocities. By combining the current position and velocity, the position update equation calculates the updated position. In the velocity update equation, the particle’s highest positions are incorporated, and the swarm’s most optimal position is determined globally.

Position Update Equation:

Yi(t+1)=Yi(t)+Xi(t+1) (14)

Velocity Update Equation:

Xi(t+1)=z*Xi(t)+D1*t1*(Pi(t)‐Xi(t))+D2*t2*(G(t)‐Xi(t)) (15)

Where,

- Y(i,t) position t at which particle i is currently located.
- X(i,t) at time t, represents the particle’s current velocity.
- z represents the particle’s inertia weight, which controls the particle’s influence on its previous velocity. There was a range of 0.9 to 0.2 inertia weight in this study
- D1 and D2 are the acceleration coefficients that control the influence of the particle’s individual best (Pi(t)) and the global best (G(t)), respectively. Both parameters are set to 2.
- t1 and t2 are random numbers between 0 and 1.

The inertia weight, denoted as z, regulates the influence of the particle’s previous velocity in the PSO process. In this study, an inertia weight range of 0.9 to 0.2 was chosen to achieve a trade-off between exploration and exploitation. A higher inertia weight (e.g., 0.9) facilitates increased exploration, enabling particles to explore a broader solution space. Conversely, a lower inertia weight (e.g., 0.2) promotes exploitation, encouraging particles to converge towards the currently identified optimal solution. The PSO algorithm is balanced between exploration and exploitation, owing to this range selection.

#### 6.3.4. Update individual and global best

An algorithm for PSO was used in this study. The number of iterations/generations cannot exceed 100 before the process is terminated.

#### 6.3.5. Termination criteria

A termination condition exists after each iteration of the PSO algorithm. A maximum of 100 iterations or generations was used as the termination criterion in this study. It is possible to incorporate two additional termination criteria into the optimization process to improve its reliability and credibility.

The algorithm terminates when a predefined threshold of difference between the objective function values of consecutive iterations is reached, as defined by the Convergence Criterion. A stable and satisfactory solution was achieved because of the optimization process.

Secondly, the Solution Stability Criterion can be incorporated, where the algorithm halts when the solution remains unchanged for a specified number of consecutive iterations. This indicates that further iterations are unlikely to yield significant improvements, suggesting that the algorithm provides a reliable solution.

#### 6.3.6. Output of the result

After the PSO algorithm terminates, the values of T2, t2, and € corresponding to the global best position are obtained. A heat exchanger with these values achieves the highest grey relational grade possible.

Using PSO for ANN parameter optimization improved GRA prediction accuracy was achieved for heat exchangers using a PSO algorithm. By iterating over neural network weights and biases, PSO found the most accurate combination. The GRA ranking values predicted and achieved were minimized as a result.

According to this study, the parameters used for this algorithm were 50 particles, 0.9 to 0.2 inertial weight, 2 cognitive parameters, 2 social parameters, and 1 maximum velocity.

The algorithm completed the optimization process, yielding a highly accurate prediction of the heat exchanger performance by effectively optimizing the grey relational grade. The best fitness value of 0.00036072 indicates a remarkable agreement between the predicted and target values. The PSO algorithm’s efficacy in achieving optimal results is further reinforced by the corresponding optimal position of 0.71836.

Table 6 illustrates a comprehensive comparison of the predicted and actual GRG values. The table also includes the error values, quantifying the disparity between the predicted and actual GRG values. This meticulous analysis underscores the precision and dependability of the PSO-optimized model in forecasting heat exchanger performance. The findings highlight the PSO algorithm’s potential to enhance the accuracy of predictions in various real-world applications.

**Table 6:** GRG VS predicted GRG.

	Target	Predicted	
Exp. No.	GRG	GRG	Error
1	0.51	0.51	0.0021
2	0.50	0.50	0.0006
3	0.51	0.51	0.0042
4	0.60	0.60	0.0007
5	0.53	0.53	0.0037
6	0.48	0.48	-0.0012
7	0.73	0.73	-0.0003
8	0.84	0.84	0.0004
9	0.66	0.66	-0.0009
10	0.44	0.44	0.0025
11	0.39	0.39	-0.0002
12	0.59	0.58	0.0058
13	0.55	0.54	0.0125
14	0.45	0.45	-0.0032
15	0.53	0.53	-0.0034
16	0.49	0.49	-0.0009

**Fig 16:**
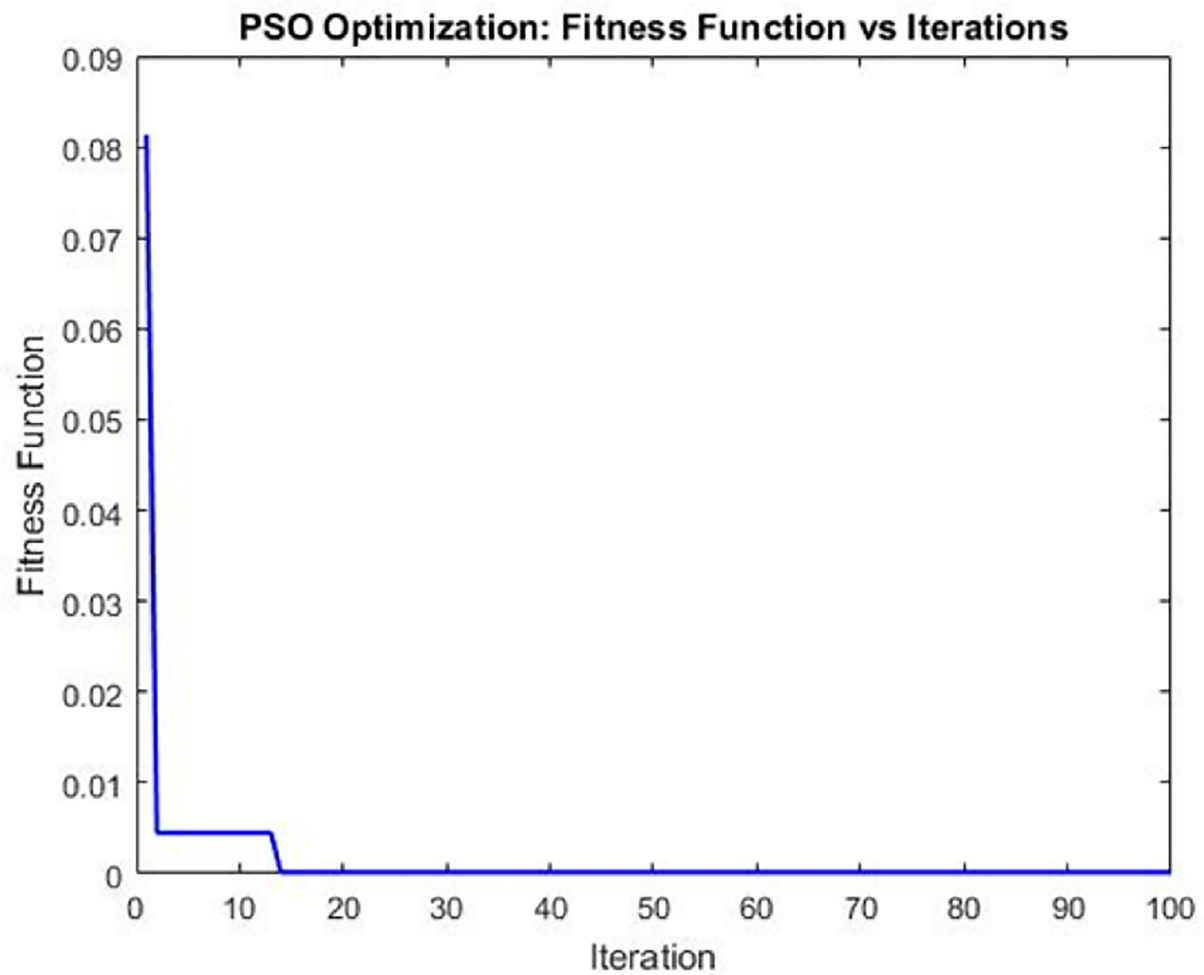
Iteration Vs fitness function.

PSO-based optimization is illustrated in Fig 16 by the relationship between Fitness Function and Iteration. It showcases the algorithm’s convergence and progress towards the optimal solution. Limited space hampers in-depth analysis and discussion. Table 7 showcases the best optimization results obtained for heat exchanger.

**Table 7:** Optimal parameters.

S.No.	mh (kg/min)	mc (kg/min)	T1 (°C)	t1 (°C)	T2 (°C)	t2 (°C)	€
1	3	3	78	30	62.98	51.41	0.646

### 6.4 Cost/Economics

In this section, we examine the financial aspects of shell and tube heat exchangers with an emphasis on researching their costs and economics. An understanding of the economic aspects is crucial for the applicability and feasibility of these heat exchange systems. Many approaches to cost estimation have been developed in the literature that depend on a variety of important variables, such as the type of apparatus, operating pressure, heat-transfer surface area, and material composition [46–48].

The stainless-steel shells and tubes were manufactured at an approximate cost of Rs. 6500/- for the construction of a specially designed shell and tube heat exchanger for laboratory research, in the Table 8 shows the consumables cost. The setup costs for the experimental apparatus were approximately Rs. 500. As a result, the approximate Rs. 7000 total investment was justified because this heat exchanger was specifically designed to satisfy highly specialized applications. When shell and tube heat exchangers are produced on a large scale in an industrial setting, they are more economically competitive for widespread commercial adoption.

**Table 8:** Equipment cost.

S. No.	Consumables	Qty.	Cost (Rupees)
1	Stainless steel pipe 90 mm Material grade SS316	01	2900.00
2	Stainless steel pipe 20 mm Material grade SS316	01	3150.00
3	Stainless steel plate (1–2 mm) Material grade SS316	01	360.00
4	Welding rods	01	100.00
5	Labour charge		500.00
Total Cost			7010.00

## 7. Conclusion

In conclusion, the hybrid optimization algorithm consisting of Gray Relational Analysis (GRE), the Neural Fitting Tool (NFTool), and Particle Swarm Optimization (PSO) has proven to be a highly effective and efficient method for improving the performance of heat exchangers in heat recovery applications. This hybrid algorithm, by combining multiple techniques, was able to find the key factors, estimate the target values, and improve the accuracy of the gray relational grade. Implementation of this algorithm on counter-flow shell and tube heat exchangers (STHE) resulted in superior performance compared to both experimental and predicted values, proving its robustness and reliability in achieving optimal performance.

The results of this study have significant implications for industry and researchers involved in heat recovery efforts. The proposed algorithm provides a practical solution to achieve these goals and provides valuable insights into heat exchanger design and operation. Additionally, the hybrid algorithm’s ability to speed up the optimization process compared to traditional methods such as the basic genetic algorithm (GA) underscores its ability to solve complex optimization problems. The combination of Particle Swarm Optimization (PSO) and GA in a hybrid approach provides a balanced and efficient solution, enabling rapid engine performance optimization in the context of this study.

## References

[1] Shabgard H., Allen M. J., Sharifi N., Benn S. P., Faghri A., & Bergman T. L. (2015). Heat pipe heat exchangers and heat sinks: Opportunities, challenges, applications, analysis, and state of the art. International Journal of Heat and Mass Transfer, 89, 138–158.

[2] Zhang Jinsong, et al. "A review of heat transfer issues in hydrogen storage technologies." (2005): 1391–1399.

[3] Florides G., &Kalogirou S. (2007). Ground heat exchangers—A review of systems, models and applications. Renewable energy, 32(15), 2461–2478.

[4] Azad AbazarVahdat, and Majid Amidpour. "Economic optimization of shell and tube heat exchanger based on constructal theory." Energy 36.2 (2011): 1087–1096.

[5] Sridharan M. (2021). Performance optimization of counter flow double pipe heat exchanger using grey relational analysis. International Journal of Ambient Energy, 1–9.

[6] Tabatabaeikia S., Mohammed H. A., Nik-Ghazali N., & Shahizare B. (2014). Heat transfer enhancement by using different types of inserts. Advances in Mechanical Engineering, 6, 250354.

[7] Tayal M. C., Fu Y., & Diwekar U. M. (1999). Optimal design of heat exchangers: A genetic algorithm framework. Industrial & engineering chemistry research, 38(2), 456–467.

[8] Liu F. B. (2008). A modified genetic algorithm for solving the inverse heat transfer problem of estimating plan heat source. International Journal of Heat and Mass Transfer, 51(15–16), 3745–3752.

[9] Feyli B., Soltani H., Haji Mohammadi R., Fallahi-Samberan M., & Eyvazzadeh A. (2022). A reliable approach for heat exchanger networks synthesis with stream splitting by coupling genetic algorithm with modified quasi-linear programming method. Chemical Engineering Science, 248, 117140.

[10] Sridharan M. (2020). Applications of artificial intelligence techniques in heat exchanger systems. In Advanced Analytic and Control Techniques for Thermal Systems with Heat Exchangers (pp. 325–334). Academic Press.

[11] Saha S. G., Sharma V., Bharadwaj A., Shrivastava P., Saha M. K., Dubey S., et al. (2017). Effectiveness of various endodontic irrigants on the micro-hardness of the root canal dentin: An in vitro study. Journal of clinical and diagnostic research: JCDR, 11(4), ZC01. 10.7860/JCDR/2017/24018.9472 PMC5449895 28571249

[12] Thanikodi S., Singaravelu D. K., Devarajan C., Venkatraman V., & Rathinavelu V. (2020). Teaching learning optimization and neural network for the effective prediction of heat transfer rates in tube heat exchangers. Thermal Science, 24(1 Part B), 575–581.

[13] El-Mihoub T. A., Hopgood A. A., Nolle L., &Battersby A. (2006). Hybrid Genetic Algorithms: A Review. Eng. Lett., 13(2), 124–137.

[14] García-Morales J., et al. "Inverse artificial neural network control design for a double tube heat exchanger." Case Studies in Thermal Engineering 34 (2022): 102075.

[15] Mamoudan Mousapour, Mobina, et al. "Hybrid neural network-based metaheuristics for prediction of financial markets: a case study on global gold market." Journal of Computational Design and Engineering 10.3 (2023): 1110–1125.

[16] Ebrahimi-Moghadam Amir, et al. "A comprehensive thermo-hydraulic analysis and optimization of turbulent TiO2/W-EG nano-fluid flow inside double-pipe heat exchangers with helical coil inserts." Journal of the Brazilian Society of Mechanical Sciences and Engineering 42.5 (2020): 232.

[17] Wang Wei, et al. "Optimal design of a double pipe heat exchanger based on the outward helically corrugated tube." International Journal of Heat and Mass Transfer 135 (2019): 706–716.

[18] Azad AbazarVahdat, and Amidpour Majid. "Economic optimization of shell and tube heat exchanger based on constructal theory." Energy 36.2 (2011): 1087–1096.

[19] Tian Zhe, et al. "Turbulent flows in a spiral double-pipe heat exchanger: optimal performance conditions using an enhanced genetic algorithm." International Journal of Numerical Methods for Heat & Fluid Flow 30.1 (2019): 39–53.

[20] Thejaraju R., and Girish K. B. "A Comprehensive Review on Design and Analysis of Passive Enhancement Techniques in Double Pipe Heat Exchanger." power 2 (2019): 2.

[21] Ebrahimi-Moghadam Amir, et al. "A comprehensive thermo-hydraulic analysis and optimization of turbulent TiO2/W-EG nano-fluid flow inside double-pipe heat exchangers with helical coil inserts." Journal of the Brazilian Society of Mechanical Sciences and Engineering 42.5 (2020): 232.

[22] Jamil Muhammad Ahmad, et al. "Exergoeconomic analysis of energy conversion systems: from fundamentals to applications." Synergy Development in Renewables Assisted Multi-carrier Systems. Cham: Springer International Publishing, 2022. 3–21.

[23] Abbasi Hamid Reza, et al. "Shape optimization of segmental porous baffles for enhanced thermo-hydraulic performance of shell-and-tube heat exchanger." Applied Thermal Engineering 180 (2020): 115835.

[24] Shahsavar Mohammad M., et al. "Constructing a smart framework for supplying the biogas energy in green buildings using an integration of response surface methodology, artificial intelligence and petri net modelling." Energy Conversion and Management 248 (2021): 114794.

[25] Thanikodi Sathish, et al. "Teaching learning optimization and neural network for the effective prediction of heat transfer rates in tube heat exchangers." Thermal Science 24.1 Part B (2020): 575–581.

[26] Gholizadeh Hadi, et al. "Fuzzy data-driven scenario-based robust data envelopment analysis for prediction and optimisation of an electrical discharge machine’s parameters." Expert Systems with Applications 193 (2022): 116419.

[27] Algarni Mohammed, Alazwari Mashhour A., and Safaei Mohammad Reza. "Optimization of nano-additive characteristics to improve the efficiency of a shell and tube thermal energy storage system using a hybrid procedure: DOE, ANN, MCDM, MOO, and CFD modeling." Mathematics 9.24 (2021): 3235.

[28] Kazi Saif R., et al. "Heat exchanger network synthesis with detailed exchanger designs—2. Hybrid optimization strategy for synthesis of heat exchanger networks." AIChE Journal 67.1 (2021): e17057.

[29] Saffarian Mohammad Reza, pour Farivar Fazel, and Mehrzad Sham. "Numerical study of shell and tube heat exchanger with different cross-section tubes and combined tubes." International Journal of Energy and Environmental Engineering 10 (2019): 33–46.

[30] Fares Mohammad, Mohammad AL-Mayyahi, and Mohammed AL-Saad. "Heat transfer analysis of a shell and tube heat exchanger operated with graphene nanofluids." Case Studies in Thermal Engineering 18 (2020): 100584.

[31] Zhan C., et al. "A hybrid approach for low-carbon transportation system analysis: integrating CRITIC-DEMATEL and deep learning features." International Journal of Environmental Science and Technology (2023): 1–14. doi: 10.1007/s13762-023-04995-6 37360563 PMC10250180

[32] Ghazikhani Adel, et al. "A smart post-processing system for forecasting the climate precipitation based on machine learning computations." Sustainability 14.11 (2022): 6624.

[33] Liang Caihang, et al. "Optimal design of an air-to-air heat exchanger with cross-corrugated triangular ducts by using a particle swarm optimization algorithm." Applied Sciences 7.6 (2017): 554.

[34] Rao R.Venkata, and Patel Vivek. "Multi-objective optimization of heat exchangers using a modified teaching-learning-based optimization algorithm." Applied Mathematical Modelling 37.3 (2013): 1147–1162.

[35] Venkatesh B.; Khan M.; Alabduallah B.; Kiran A.; Babu J.C.; Bhargavi B.; et al. Design Optimization of Counter-Flow Double-Pipe Heat Exchanger Using Hybrid Optimization Algorithm. Processes 2023, 11, 1674. doi: 10.3390/pr11061674

[36] Zhang Zichen, Ding Shifei, and Jia Weikuan. "A hybrid optimization algorithm based on cuckoo search and differential evolution for solving constrained engineering problems." Engineering Applications of Artificial Intelligence 85 (2019): 254–268.

[37] Hasanuzzaman M., Saidur Rahman, and Rahim N. A. "Effectiveness enchancement of heat exchanger by using nanofluids." 2011 IEEE Conference on Clean Energy and Technology (CET). IEEE, 2011.

[38] Guo Zengyuan Y., et al. "Effectiveness–thermal resistance method for heat exchanger design and analysis." International Journal of Heat and Mass Transfer 53.13–14 (2010): 2877–2884.

[39] Kuo Yiyo, Yang Taho, and Huang Guan-Wei. "The use of grey relational analysis in solving multiple attribute decision-making problems." Computers & industrial engineering 55.1 (2008): 80–93.

[40] Saindane Rahul P., and Wasankar K. S. "Multi-Objective Optimization of Turning Process Parameters for EN353 Material using Taguchi based Grey Relational Analysis." International Journal of Engineering and Management Research (IJEMR) 5.3 (2015): 789–796.

[41] Bademlioglu A. H., Canbolat A. S., and Kaynakli O. "Multi-objective optimization of parameters affecting Organic Rankine Cycle performance characteristics with Taguchi-Grey Relational Analysis." Renewable and Sustainable Energy Reviews 117 (2020): 109483.

[42] Tzeng Chorng-Jyh, et al. "Optimization of turning operations with multiple performance characteristics using the Taguchi method and Grey relational analysis." Journal of materials processing technology 209.6 (2009): 2753–2759.

[43] Bademlioglu A. H., Canbolat A. S., and Kaynakli O. "Multi-objective optimization of parameters affecting Organic Rankine Cycle performance characteristics with Taguchi-Grey Relational Analysis." Renewable and Sustainable Energy Reviews 117 (2020): 109483.

[44] Rao R. V., and Patel V. K. "Thermodynamic optimization of cross flow plate-fin heat exchanger using a particle swarm optimization algorithm." International Journal of Thermal Sciences 49.9 (2010): 1712–1721.

[45] Peng Hao, Ling Xiang, and Wu En. "An improved particle swarm algorithm for optimal design of plate-fin heat exchangers." Industrial & Engineering Chemistry Research 49.13 (2010): 6144–6149.

[46] Kumar Harikirupakar Kishore, Nagaraj Prajwal, and Uday Rohan Halageri. "Design Optimization of a Shell and Tube Heat Exchanger." MAE 598 Design Optimization (2016).

[47] Jarić Marko, et al. "Total costs of shell and tube heat exchangers with concentric helical tube coils." Thermal Science 23.6 (2019): 3661–3673.

[48] Cotrim Syntia Lemos, et al. "Parameters for cost estimation in shell and tube heat exchangers network synthesis: A systematic literature review on 30 years of research." Applied Thermal Engineering 213 (2022): 118801.

